# Influence of Fluid Ingestion on Heart Rate, Cardiac Autonomic Modulation and Blood Pressure in Response to Physical Exercise: A Systematic Review with Meta-Analysis and Meta-Regression

**DOI:** 10.3390/nu15214534

**Published:** 2023-10-26

**Authors:** Andrey A. Porto, Cicero Jonas R. Benjamim, Andressa Crystine da Silva Sobrinho, Rayana Loch Gomes, Luana A. Gonzaga, Guilherme da Silva Rodrigues, Luiz Carlos Marques Vanderlei, David M. Garner, Vitor E. Valenti

**Affiliations:** 1 Department of Movement Sciences, São Paulo State University, UNESP, Presidente Prudente 19060-900, SP, Brazil; luana.gonzaga@unesp.br (L.A.G.); vitor.valenti@unesp.br (V.E.V.); 2 Department of Internal Medicine, Ribeirão Preto Medical School, University of São Paulo, Ribeirão Preto 14049-900, SP, Brazil; jonasbenjamim@usp.br (C.J.R.B.); andressa.sobrinho@usp.br (A.C.d.S.S.); guirodrigues@usp.br (G.d.S.R.); 3 Department of Nutrition, Faculty of Health Sciences, Federal University of Grande Dourados, Dourados 79804-970, MS, Brazil; rayanagomes@ufgd.edu.br; 4 Department of Physiotherapy, Sao Paulo State University, UNESP, Presidente Prudente 19060-900, SP, Brazil; lcm.vanderlei@unesp.br; 5 Cardiorespiratory Research Group, Department of Biological and Medical Sciences, Faculty of Health and Life Sciences, Oxford Brookes University, Oxford OX3 0BP, UK; davidmgarner1@gmail.com

**Keywords:** water intake, electrolyte balance, dehydration, heart rate control, cardiovascular physiology, autonomic nervous system

## Abstract

A systematic review was undertaken to investigate the involvement of hydration in heart rate (HR), HR variability (HRV) and diastolic (DBP) and systolic (SBP) blood pressure in response to exercise. Data synthesis: The EMBASE, MEDLINE, Cochrane Library, CINAHL, LILACS and Web of Science databases were searched. In total, 977 studies were recognized, but only 36 were included after final screening (33 studies in meta-analysis). This study includes randomized controlled trials (RCTs) and non-RCTs with subjects > 18 years old. The hydration group consumed water or isotonic drinks, while the control group did not ingest liquids. For the hydration protocol (before, during and after exercise), the HR values during the exercise were lower compared to the controls (−6.20 bpm, 95%CI: −8.69; −3.71). In the subgroup analysis, “water ingested before and during exercise” showed lower increases in HR during exercise (−6.20, 95%CI: 11.70 to −0.71), as did “water was ingested only during exercise” (−6.12, 95%CI: −9.35 to −2.89). Water intake during exercise only revealed a trend of avoiding greater increases in HR during exercise (−4,60, 95%CI: −9.41 to 0.22), although these values were not significantly different (*p* = 0.06) from those of the control. “Isotonic intake during exercise” showed lower HRs than the control (−7.23 bpm, 95% CI: −11.68 to −2.79). The HRV values following the exercise were higher in the hydration protocol (SMD = 0.48, 95%CI: 0.30 to 0.67). The values of the SBP were higher than those of the controls (2.25 mmHg, 95%CI: 0.08 to 4.42). Conclusions: Hydration-attenuated exercise-induced increases in HR during exercise, improved autonomic recovery via the acceleration of cardiac vagal modulation in response to exercise and caused a modest increase in SBP values, but did not exert effects on DBP following exercise.

## 1. Introduction

Fluid loss during physical exercise poses challenges to the cardiovascular system [1]. Plasma osmolality increases and sympathetic modulation of the heart is intensified to elevate cardiac output [2]. Simultaneously, there is an upsurge in vasopressin secretion, which increases water reabsorption via the renal tubules and, together with the renin–angiotensin–aldosterone system, increases levels of vasoconstriction [3]. The hemodynamic and hormonal systems work concurrently to maintain blood pressure (BP) at acceptable levels and prevent dehydration [4,5].

Given the impact of fluid loss on the body’s physiology, fluid replacement through and following exercise was a concern in several studies. Controlled fluid ingestion prior to physical exercise and fluid replacement during and after physical exercise is critical to avoid dehydration [6]. Alternatively, excessive fluid replacement without electrolyte control can lead to hyperhydration [6]. In longitudinal observational studies, dehydration and hyperhydration conditions are linked with increased adverse cardiovascular events and mortality [7]. Dehydration is linked to increases in the risks inherent in periods of exercise and recovery [1,2,8] and can negatively affect the cardiovascular [1] and autonomic systems [9].

The increase in cardiovascular stress in response to the greater thermal effort generated by dehydration leads to a reduction in systolic volume, which seems to be related to a reduction in central venous pressure, prompting an increase in baroreflex sensitivity and, so, greater sympathetic activity, which impairs autonomic recovery. Additionally, dehydration reduces heat tolerance, leading to lower exercise safety and performance [9]. However, it is unknown whether subjects who are persistently exposed to physical exercise (e.g., professional and amateur athletes and other physically active subjects) experience such repercussions, with undesirable outcomes for their cardiovascular systems in the long term [10].

The analysis of cardiovascular behavior during exercise and the recovery phase is a valuable instrument for forecasting risk and sudden cardiac death in situations that enforce physiological stress, such as physical exercise [11,12,13,14,15]. The measurement of heart rate (HR) and HR variability (HRV), which permits the study of cardiac autonomic modulation, is regarded as a practical and reliable method for analyzing the outcomes of cardiovascular system interventions in response to physical exercise [16,17,18,19,20,21]. Furthermore, BP analyses with these variables create an understanding of hemodynamic control and, consequently, a more accurate analysis of global cardiovascular function [12,21].

Several studies have explored the effects of fluid ingestion on these parameters. However, by reason of conflicting results and small sample sizes (cohorts), there is no consensus on the outcomes achieved amongst the primary studies. So far, effect estimators have not been completed to evaluate the cardiovascular and autonomic repercussions of the effects of fluid replacement or non-replacement through exercise. Furthermore, in this field of study, the fluid ingestion time is heterogeneous among the studies. It can occur only before, during and after exercise—or a combination of these periods. Therefore, an in-depth analysis of previous studies is required to assess how the time of fluid ingestion influences the cardiovascular and autonomic systems. The comprehension of the real effects of hydration status and dehydration on the clinical and standard health parameters of physical activity is required from practitioners to enable cardiovascular prevention strategies. With the aim of resolving these matters, we performed a systematic literature review and applied a meta-analytic model to study the consequences of fluid or non-fluid ingestion and replacement for HR, cardiac autonomic modulation and BP. Furthermore, we applied meta-regression models to elucidate the extent to which specific variables (e.g., exercise duration, temperature and body weight loss) describe changes in cardiac performance throughout physical exercise.

## 2. Materials and Methods

### 2.1. Registration

The review was reported according to the recommendations of the Preferred Reporting Items for Systematic Reviews and Meta-Analyses (PRISMA) [22] and listed in the PROSPERO database (CRD42022347000).

### 2.2. Search Strategy and Study Selection

The searches were performed on 14 June 2022—and actualized before the peer-review process on 14 February 2023—via EMBASE, MEDLINE (PubMed), Cochrane Library, CINAHL, LILACS and Web of Science databases with the submission of the keywords “Heart Rate Variability” OR “Autonomic recovery” OR “Vagal Reactivation” OR “Heart rate recovery” OR “Blood Pressure” OR “Heart Rate” AND “Hydration” OR “Fluid replacement” OR “Water intake” OR “Rehydration” OR “Isotonic beverages” OR “Sports beverages” AND “Exercise” OR “Physical activity”. No restrictions were applied to the studies’ languages, dialects (see search strategy in Supplementary Materials) or years of publication.

All articles approved were exported to the Rayyan QCRI program (Qatar Computing Research Institute, Qatar) to eliminate duplicates. The studies were screened using the Rayyan program by reading the title and abstract. The eligibility stage was completed by two independent reviewers (AAP and CJRB) by reading the entire articles. An additional reviewer was asked to make a decision (VEV) if there was a discrepancy about a study.

We identified studies published from the start of the database up until 14 February 2023. For inclusion, the articles were required to achieve all the criteria described below: RCTs or non-RCTs design and participants older than 18 years. The intervention group consisted of individuals who orally ingested water or isotonic beverages, and the control group of individuals who did not ingest liquids (in only three articles, the control group consumed 50 mL of water; in one article, consumption occurred before exercise and in two articles, it occurred later). Oral hydration could be imposed before, during or directly after the exercise sessions’ conclusion (subgroups have been created to analyze the results of each specific moment of liquid intake). There are no established criteria regarding the type of physical exercise and their length or modalities, and the details of characteristics of the study population in the articles were described exactly as in the main table results (Table 1). The studies evaluated HR, BP or the outcomes of HRV measured during or after the exercise regimes; the studies were not excluded if they logged one but not all variables.

### 2.3. Data Extraction

Data about the author, study design, study participant features, interventions and exercise protocols of the respective studies were taken out from primary studies and presented in Table 1. Absent datasets were requested by contacting the corresponding study authors. This stage was completed independently by two reviewers (CJRB and ACS). When the author’s correspondent did not respond, the Web Plot Digitizer^®^ was applied to extract the data presented in graphs. The post-intervention data was charted as mean and standard deviations (SD). Values giving “standard error” or “confidence intervals’’ (CI) in the primary studies were converted to SD. Data were extracted from the studies regarding the hydration time, and the following criteria were necessary to classify what each sub-group represents:

1. Water intake prior to and during exercise: studies that provided participants with water ingestion at least two hours prior to starting the exercise and maintained the intervention through exercise;
2. Water intake throughout exercise: studies that completed water ingestion just during the exercise;
3. Water intake after exercise: studies that enforced water ingestion only after exercise (before and during exercise, the participants did not ingest water or isotonic drinks);
4. Isotonic during exercise: studies that achieved hydration with isotonic drinks just during exercise;
5. Isotonic after exercise: studies that completed isotonic drinks ingestion just after exercise.

### 2.4. Assessment of the Risk of Bias

The analysis of bias was accomplished using risk of bias tools, which originated in the Cochrane organization [58] using the Review Manager program (RevMan 5.4.1). Risk of bias is a tool that was founded on the domains [59], and its evaluation was separated into seven fields: “Random sequence generation”, “Allocation concealment”, “Blinding of participants and personnel”, “Blinding of outcome assessment”, “Incomplete outcome data”, “Selective reporting”, and “Other Bias”. The classification was divided into three direct responses: low risk, unclear risk, and high risk. Our deductions were founded on the table developed by Carvalho et al. [59]: “Reviewers’ judgment and criteria for judgment”. Two independent authors completed the risk of bias analysis (AAP and CJRB). One more reviewer (VEV) was consulted if there were any contradictions in their verdicts.

### 2.5. Meta-Regression Analysis

A random effect meta-regression was completed to assess the relationship between the significant estimated effect sizes with potential moderator covariates. Data was completed via the Comprehensive Meta-analysis 3.3.070 (Biostat Inc.^®^, Englewood, NJ, USA) program.

### 2.6. Publication Bias

Potential publication biases were considered using a visual inspection of Begg’s funnel plot asymmetry, Begg’s rank correlation test, and Egger’s weighted regression test. Duval and Tweedie’s “trim and fill” method was imposed to adjust the analysis for the effects of publication biases [60]. Moreover, data was formed using the Comprehensive Meta-analysis 3.3.070 (Biostat Inc.^®^) program.

### 2.7. GRADE (Levels of Evidence)

The Grades of Recommendation, Assessment, Development, and Evaluation (GRADE) Working Group (GRADE Working Group, 2004) was enforced to study the certainty of the evidence, in addition to the study design of non-randomized (weak evidence) or randomized trials (strong evidence). Study quality (detailed study methods and execution) and significant limitations were secondarily considered in the strength of evidence analysis [61]. The summary of achievements was performed using GRADEpro GDT version 4^®^ (McMaster University, Hamilton, ON, Canada). The recommendation was created for each key variable investigated (HR, HRV, SBP, and DBP), and it was established that a specific approval would be made when a sub-group analysis has a number equal to or greater than four studies. The level of evidence is presented in Table 2.

### 2.8. Qualitative Analysis (Systematic Review)

A descriptive synthesis was instigated to describe detailed data on how each study was completed. The details for each study were introduced using texts and tables. The results of the individual qualitative analysis per study were finalized by analyzing the HRV index, HR, and BP for the hydration or control protocols.

### 2.9. Quantitative Analysis (Meta-Analysis)

In the meta-analysis, we presented BP (mmHg), HR (beats per minute), and HRV index (milliseconds, natural logarithm and normalized units) values.

The studies that were introduced in the meta-analysis more than once to analyze the same outcome (e.g., HR during exercise) had their sample number divided by the number of times they were entered into the meta-analysis (the same allocation sample group) to avoid double counting. The material required to construct the meta-analysis was the period post intervention. We assumed the criterion for extracting HR data from the last dataset presented during exercise, owing to the effect that dehydration during exercise is cumulative with regards to the beginning. To assess HRV outcomes, the only indexes which represent vagal modulation considered for inclusion were RMSSD—the square root of the mean of the sum of the squares of differences between adjacent heartbeats (RR intervals) in milliseconds (ms) and natural logarithm (ln); and SD1—standard deviation of instantaneous beat-to-beat variability derived from the Poincaré Plot analysis. In the frequency domain, the high frequency (HF) spectral power (0.15 Hz to 0.4 Hz) was calculated in normalized units (n.u) and absolute units (ms). The vagal indices described above were chosen as they are more widely used in this research domain and situations mediated by physical exercise, as they have greater validity as a result of their accuracy [62]. Regarding the outcomes for BP, we included systolic and diastolic values. The BP and HRV data from the initial data were presented between groups in post-exercise recovery. In this case, the studies that assessed exercise responses and those that evaluated recovery were detached.

For the HR, SBP, and DBP analysis, we imposed a random effect and mean difference (MD) model to quantify the effect size. In the HRV analysis, we applied a random effect and standardized mean difference (SMD) model because of mixed indexes and measurement units included in the analysis. Heterogeneity was computed using the I^2^ statistic, where a number greater than 50% (>50%) was considered to indicate substantial heterogeneity between the tests [63], for the values of “95% CI” and “test for overall effect size,” values of *p* < 0.05 (or <5%) were expected as significant differences. We enforced a random effects model since that is a more conservative method that permits that the heterogeneity of the study may vary beyond chance, providing further generalizable results [58]. All data was formed using the Review Manager Program (RevMan 5.4.1).

## 3. Results

### 3.1. Description of Studies

**Figure 1:**
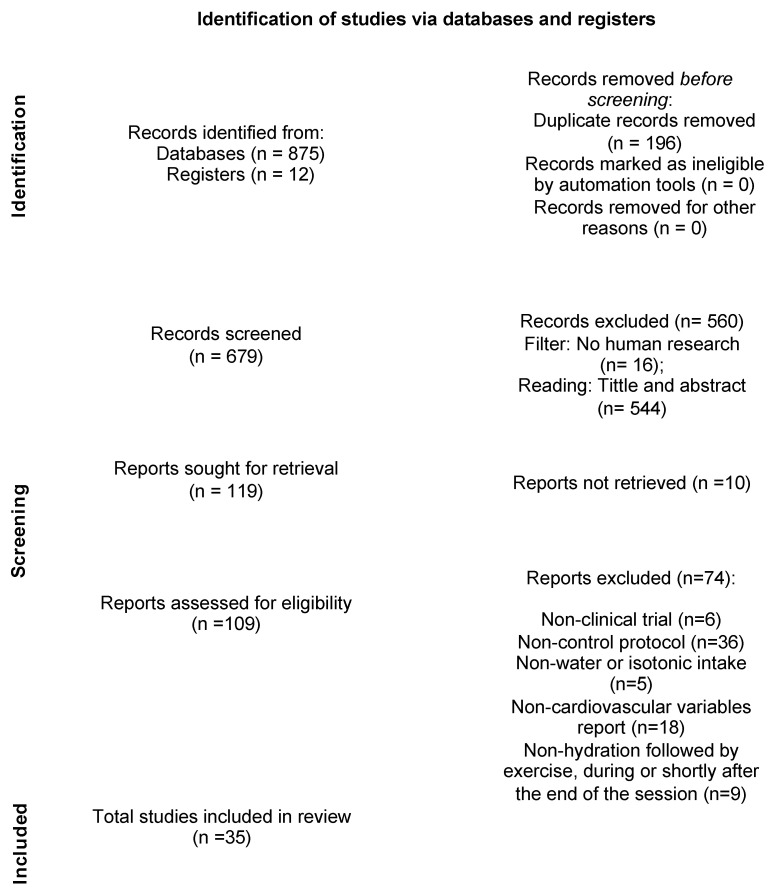
Flowchart PRISMA.

A total of 35 studies [22,23,24,25,26,27,28,29,30,31,32,33,34,35,36,37,38,39,40,41,42,43,44,45,46,47,48,49,50,51,52,53,54,55,56,57] published between 1991 and 2022 were included in the qualitative analysis (systematic review) and the characteristics of the studies are provided in Table 1. Thirty-three studies were included in the quantitative synthesis (meta-analysis). Amongst them, 24 studies assessed HR during exercise [22,23,24,26,27,28,29,34,35,38,40,42,43,45,46,47,48,50,51,52,54,55,56,57], 11 studies analyzed HRV recovery [28,31,32,33,34,36,39,40,51,53,54] and 7 analyzed SBP and DBP throughout recovery [26,30,31,37,41,52,55]. The search process and selection step details are established in the PRISMA protocol flow diagram (Figure 1).

The majority of the judged studies were undertaken with physically active individuals. Only four articles required physically inactive individuals [24,26,30,41]. The majority of studies were concerned with maintaining tests completed at the same time of day to standardize circadian influences. Some studies described the specified time of day throughout the morning [29,34,39,43,44,45] or afternoon [26,30,33,36,37,40,51,52,54,55].

The studies kept standardization of the instructions prior to the test, recommending them to refrain from alcoholic beverages or caffeine [22,23,25,27,28,30,31,33,34,36,37,39,40,41,46,49,52,53,54,55,56] and not undertake any physical exercise at least 24 hours before the tests or adopt a usual exercise regimen [22,23,24,25,27,28,30,31,32,33,34,35,36,37,40,41,43,44,46,48,49,50,51,52,53,54,55,56,57]. Regarding the meals prior to the implementation of the experimental protocol, some studies adopted overnight fasting [25,38,42,43,44,46], light meals, or consuming a typical diet with an interval of a few hours before the tests [22,23,26,27,28,30,32,33,35,36,37,39,41,52,54,55,56,57].

Some studies were included more than once in the statistical analysis because of the distinct characteristics of the studied groups (HR results). Berkulo et al. [22] enforced three groups: no fluid ingestion during pre-exercise and ad libitum fluid intake all through training; sufficient fluid intake during the pre-exercise protocol to avoid any variation in body mass; and no fluid intake during training.

Zacharakis et al. [23] implemented two cohorts to evaluate water intake during exercise, one group had spinal cord injuries, and the other group had no spinal cord injuries; both groups consumed 85% of sweat losses that were replaced by drinking water. Hasegawa et al. [24] involved nine participants under two different conditions: without water intake and water intake at 5 min intervals, in which the amount of fluid lost in the sweat test was required for water intake. The inclusion of this article twice was attributable to a change in exercise, one session was at 60% of maximal oxygen consumption (VO2_max_), and the other was at 80% of VO2_max_.

In the study led by McConnell et al. [27], the author submitted the participants to two conditions regarding the water intake during the exercise so that the first appropriate rate of liquid intake was equal to 100% of the body mass loss and the second was 50%, with the participants being their own control in both situations. In the research directed by Armstrong et al. [35], heat stress tests varied in pretest hydration (2 euhydrated and 2 hypo hydrated trials) and water intake during exercise (2 water ad libitum and 2 no water trials). Water intake was achieved according to the total body sweat rate, which was calculated using the body mass loss. Ribeiro et al. [44] assessed normotensive and hypertensive groups through the water intake condition during exercise. Melo-Marins et al. [45] enforced the identical group for two different conditions of water intake during exercise. The first condition was hydration with customized volume, where water was consumed founded on the individual sweat rate every 10 minutes of exercise. Then, in the second condition water intake was ad libitum.

Tripette et al. [48] operated on two distinct sets of groups: a group of patients with sickle cell trait and a group without the genetic condition. Vanderlei et al. [55] applied the identical group in two different conditions: water and isotonic intake during exercise. The amount of water or isotonic fluid directed during exercise and recovery was found in body weight changes logged before and after exercise. Lynn et al. [56] evaluated three groups: no fluid intake during exercise; no fluid intake during warm exercise; and sufficient fluid intake during exercise protocol to circumvent any change in body mass. Montain et al. [57] subjected participants to three hydration conditions of isotonic intake before and during exercise.

Moreno et al. [37] compared isotonic intake in two circumstances: during and following physical exercise. McDermott et al. [25] presented three different forms of hydration: oral, intravenous, a combination of oral and intravenous, and no water consumption (control). Throughout the results of SBP, Vanderlei et al. [55] needed to be entered more than once during the statistical analysis. This was essential due to the descriptions above.

### 3.2. Qualitative Analysis

In total, 20 studies assessed HR response during physical exercise between adaptation protocols and control conditions: 16 studies reported a diminution in HR increases through physical exercise in the fluid ingestion protocol compared to the non-fluid ingestion protocol [22,23,24,28,29,31,33,35,38,42,44,49,50,52,56,57], and 5 studies revealed no significant differences between the protocols [25,26,43,54,55]. All studies that evaluated HRV during exercise established that hydration had a slight influence on vagal withdrawal during physical exercise [31,32,33,37].

SBP and DBP responses during physical exercise exhibited conflicting results between studies. Four studies identified a significantly higher SBP and DBP compared to the control group during physical exercise [41,43,54]. The other studies did not support the stated results. Mendonça et al. (2013) [43] found no significant differences between groups, and the other four studies that evaluated SBP and DBP revealed less significant increases during exercise in the hydration protocol [28,44,52,55].

During the recovery investigation, 13 studies confirmed an optimization of HR recovery to baseline following exercise [25,28,30,31,32,33,34,36,45,48,50,54,55], and the others revealed no significant difference between the protocols [26,41,46]. All studies that enforced hydration protocols constantly improved HRV recovery after physical exercise [31,32,33,34,36,39,40,51,53,55].

In the hydration protocol, seven studies stated higher values in SBP and DBP during recovery after exercise compared to the control [26,41,43,44,50,52,54]. One study exhibited lower SBP and DBP in fluid ingestion protocol equated to the control [25], and the others revealed no significant difference in variables amongst interventions [30,31,32,55]. Patients with arterial hypertension had increases in HR, SBP, and DBP during exercise in the control protocol [44]. These data highlight the importance of fluid ingestion during exercise to this cohort to evade further cardiovascular stress during effort. In athletes presenting with spinal cord injuries, the hydration protocol attenuated the HR increase during exercise in contradiction to the control protocol [23]. In patients with coronary artery disease, fluid ingestion improved the post-exercise cardiac autonomic vagal reactivation [33]. Yet, the fluid ingestion applied to subjects with pure autonomic failure did not establish improvement in their cardiovascular responses during and in recovery from exercise [26].

### 3.3. Analysis of the Risk of Bias

**Figure 2:**
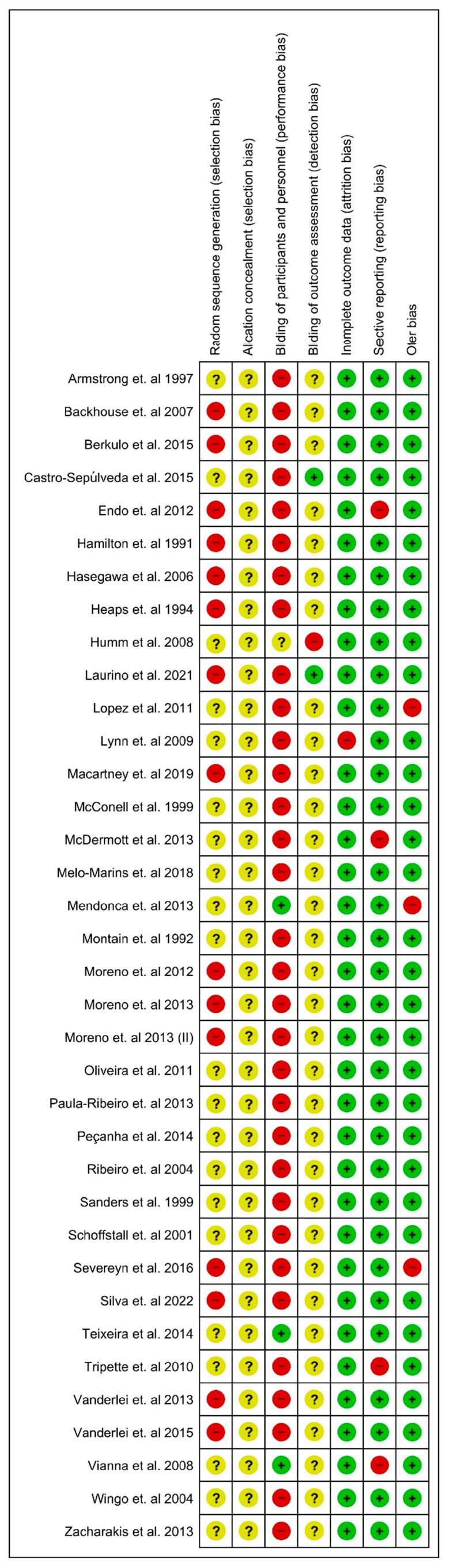
Cochrane risk of bias tool [22,23,24,25,26,27,28,29,30,31,32,33,34,35,36,37,38,39,40,41,42,43,44,45,46,47,48,49,50,51,52,53,54,55,56,57].

The risk of bias in the 35 involved studies was variable. We have summarized the results in Figure 2 with further details about the risk of bias in the included studies reported in the supplementary document “Review Authors” judgments about each risk of bias item for each included study (Supplementary Materials). Allocation: Nineteen studies (54%) enforced procedures for generating the randomization sequence but did not provide particulars of how the process was completed. Thirty-four studies (100%) did not report on methods to conceal allocation. Blinding: Participants and assessors blinding was not possible in 33 (94%) studies because of the nature of the intervention. Thirty-three studies (94%) did not report on methods to blinded assessors of outcomes. Incomplete outcome data: Thirty-four studies (97%) had the whole outcome data. Selective reporting: Thirty-one studies (89%) were free of selective outcome reporting. Other potential sources of bias: Thirty-two studies (91%) were free of other bias (see Figure 2 and ‘Review authors’ judgments about each risk of bias item for each included study in Supplementary Materials).

### 3.4. Quantitative Analysis

For the HR, SBP and DBP results, we imposed a random effect and mean difference (MD) model to quantify the effect size. In the HRV analysis, we enforced a random effect and standardized mean difference (SMD) model on account of mixed indexes included in the analysis. In both studies, the black diamond dimension represents the 95% CI. In relation to HR, a negative effect indicates decreased HR in the hydration group compared to the control. Concerning SBP and DBP, a positive effect indicates increased BP in the hydration group related to control. As regards HRV, a positive effect indicates a greater vagal influence on HRV in the hydration group compared to the control.

#### 3.4.1. Heart Rate

**Figure 3:**
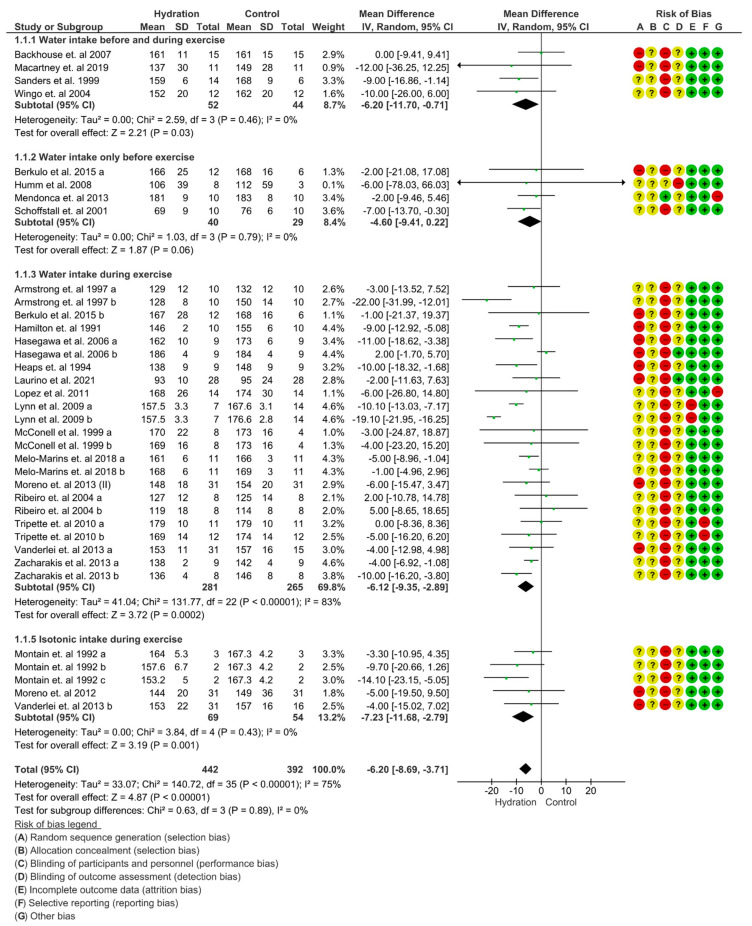
Meta-analysis for overall effects of fluid replacement on heart rate (bpm) during exercise [22,23,24,26,27,28,29,34,35,38,40,42,43,45,46,47,48,50,51,52,54,55,56,57].

A critical decrease was detected in the “test for overall effect”: subtotal was −5.28 bpm (95% CI: −6.77; −3.79), *p* < 0.001 and heterogeneity of 5%. In the sub-group “water intake before and during exercise”, significant changes were detected in the “test for overall effect”: subtotal was −6.20 bpm (95% CI: −11.70; −0.71), *p* = 0.03 and heterogeneity of 0%. In the sub-group “water intake during exercise’’, significant changes were experienced in the “test for overall effect”: subtotal was −6.12 bpm (95% CI: −9.35; −2.89), *p* < 0.001 and heterogeneity of 53%. In the sub-group “water intake only before exercise” (*p* = 0.06) and “isotonic intake during exercise”, significant changes were recognized in the “test for overall effect”: subtotal was −7.23 bpm (95% CI: −11.68; −2.79), *p* = 0.001 and heterogeneity of 0%. No differences were detected between groups (*p* = 0.83), and I^2^ = 0%. (Figure 3). The GRADE quality of evidence for this result was high.

#### 3.4.2. Heart Rate Variability

**Figure 4:**
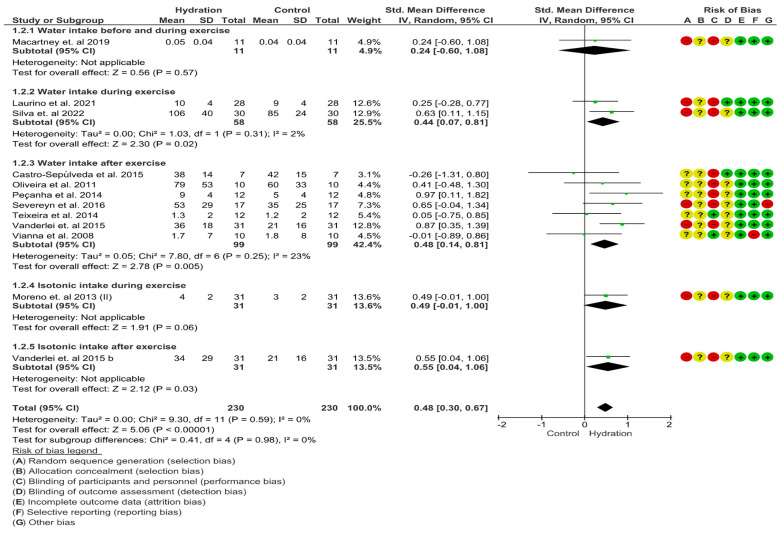
Meta-analysis for overall effects of fluid replacement on heart rate variability (standard mean difference) following exercise [28,31,32,33,34,36,39,40,51,53,54].

An important escalation was noticed in the “test for overall effect,” revealing a subtotal SMD = 0.48 (95% CI: 0.30; 0.67), *p* < 0.00 and heterogeneity of 0%. In the sub-group “water intake during exercise”, significant changes were detected in the “test for overall effect,”: subtotal SMD = 0.44 (95% CI: 0.07; 0.81), *p* = 0.02 and heterogeneity of 2%. In the sub-group “water intake after exercise”, significant changes were recognized in the “test for overall effect”: subtotal SMD = 0.48 (95% CI: 0.14; 0.81), *p* < =0.001 and heterogeneity of 23%. In the sub-group “isotonic intake after exercise”, significant changes were recognized in the “test for overall effect”: subtotal SMD = 0.55 (95% CI: 0.04; 1.06), *p* = 0.03 and heterogeneity was not applicable. In the sub-group “water intake before and during exercise” (*p* = 0.57) and “isotonic intake during exercise” (*p* = 0.06), no significant changes were observed. No differences were experienced between groups (*p* = 0.98), and I^2^= 0% (Figure 4). The GRADE quality of evidence for this result was moderate.

#### 3.4.3. Blood Pressure

**Figure 5:**
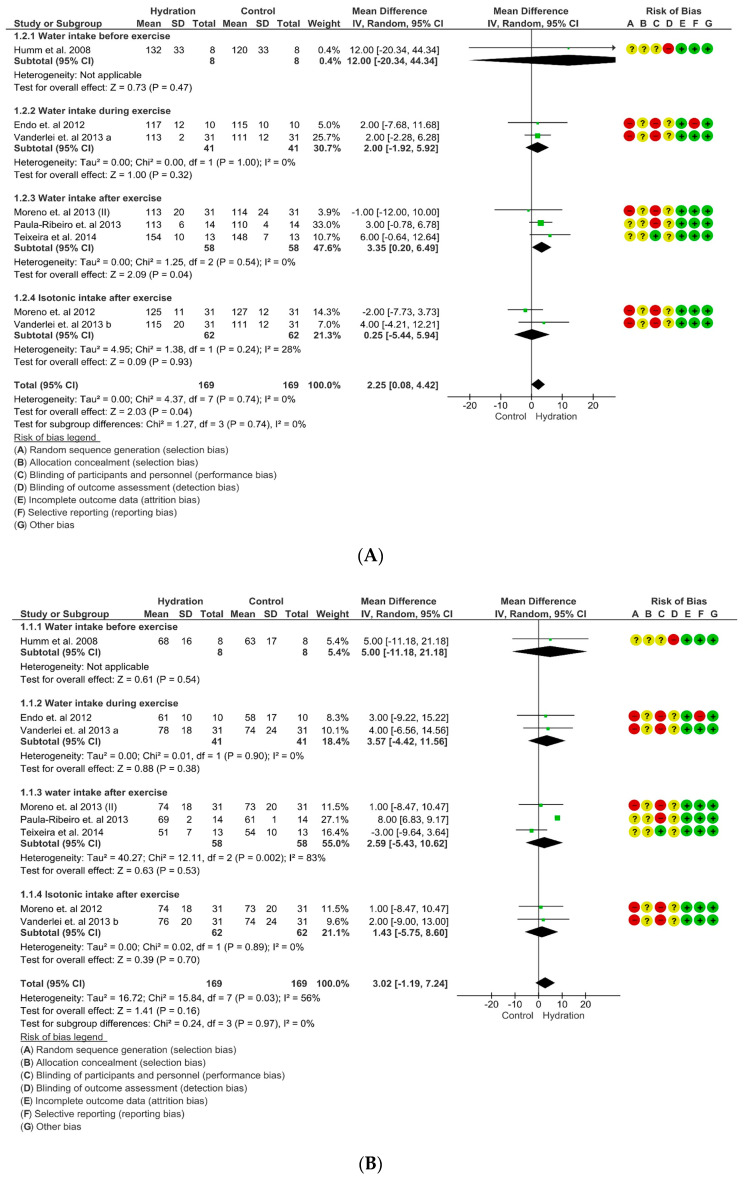
(**A**) Meta-analysis for overall effects of fluid replacement systolic blood pressure (mmHg) following exercise. (**B**) Meta-analysis for overall effects of fluid replacement diastolic blood pressure (mmHg) following exercise [26,30,31,37,41,52,55].

Concerning SBP, higher values were detected in the “test for overall effect,” where we discovered a subtotal = 2.25 mmHg (95% CI: 0.08; 4.42), *p* = 0.05 and heterogeneity of 0%. In the sub-group “water intake after exercise” values, significant changes were observed in the “test for overall effect”: subtotal = 3.35 mmHg (95% CI: 0.20; 6.49), *p* = 0.04 and heterogeneity of 0%. In the sub-group “water intake before exercise” (*p* = 0.47), “water intake during exercise” (*p* = 0.32) and “isotonic intake after exercise” (*p* = 0.93) no significant changes were detected. No differences were observed between groups (*p* = 0.74), and I^2^ = 0%. (Figure 5A). The GRADE quality of evidence for this result was low.

With regards to DBP, no difference was detected. In the “test for overall effect,” we revealed a subtotal = 3.02 mmHg (95% CI: −1.19; 7.24), *p* = 0.16 and heterogeneity of 56%. In the sub-group, “water intake before exercise” (*p* = 0.54), “water intake during exercise” (*p* = 0.38), “water intake after exercise” (*p* = 0.53) and “isotonic intake after exercise” (*p* = 0.89) no significant changes were observed. No differences were observed between groups (*p* = 0.97), and I^2^ = 0%. (Figure 5B). The GRADE quality of evidence for this result was low.

### 3.5. Meta-Regression

**Figure 6:**
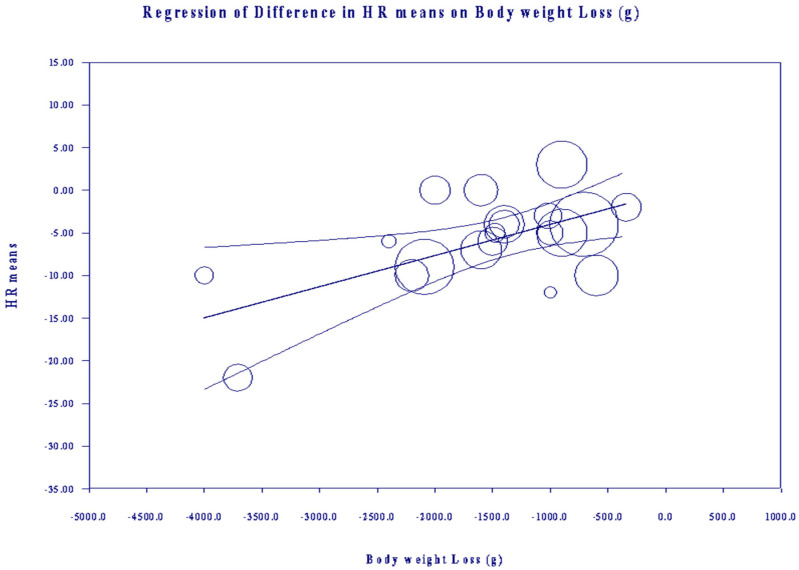
Meta-regression for the effect of body weight loss (continuous variable) on heart rate during exercise.

Meta-regression bubble plots of the relationship between mean changes in HR and “body weight loss” (Figure 6). On the “X” axis, the more negative the number, the greater the weight subtraction throughout the exercise. On the “Y” axis, the more negative the number, the lower the HR. Each circle size is inversely proportional to the variance of change. Coefficient: −00061; standard error (SE): 0.0025; Z-value: −2.47; and *p*-value: 0.01 (CI 95% −0.0013 to 0.0109) (see the meta-regression data in Supplementary Materials). Other covariables were studied independently. We noted that “exercise time”, “temperature”, “BMI”, “mean age”, “exercise intensity” and “allocation” offered no relationship with HR (see “meta-regression data 2” in Supplementary Materials). With regards to HRV, meta-regression was unnecessary due to the heterogeneity value (0%). Consistent with the Cochrane Handbook, we did not perform a meta-regression of BP as a result of the small number (n < 10) of studies that offered outcomes of SBP and DBPs [58].

### 3.6. Publication Bias

**Figure 7:**
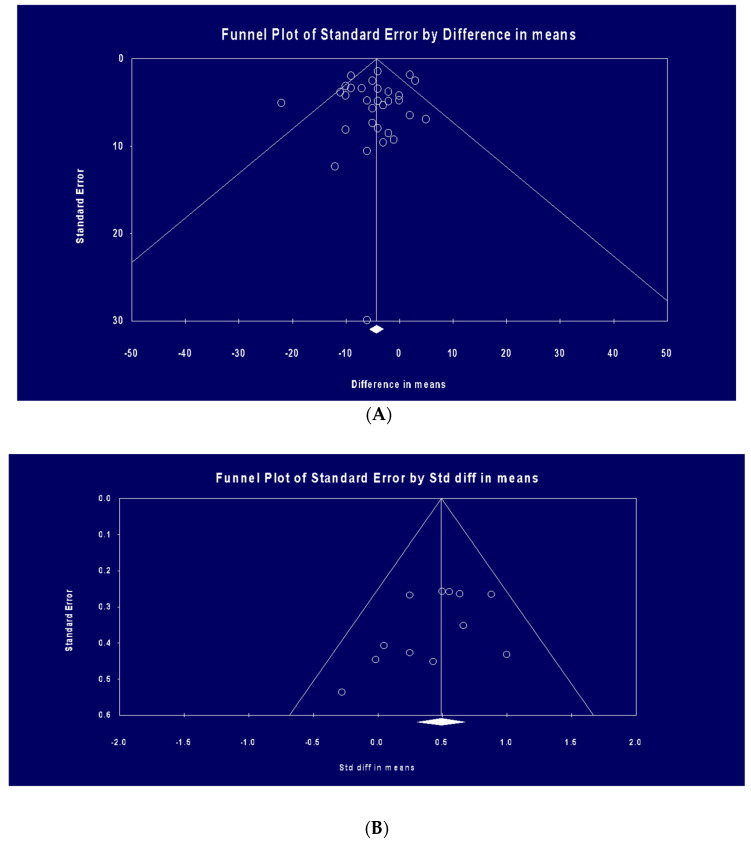
(**A**) Funnel plot of standard error by difference in means (studies analyzing HR during exercise). (**B**) Funnel plot of standard error by standard difference in SDM (studies analyzing HRV recovery following exercise).

The funnel plot defined publication biases in the studies included in the meta-analysis (HR during exercise and HRV on recovery) (Figure 7A,B)

The funnel plots of standard error by effect size (MD) were asymmetric (Figure 7A), signifying potential publication biases in the meta-analysis of the hydration effect on the HR index. The existence of publication biases was not confirmed by Egger’s linear regression (*p* = 0.53). Begg’s rank correlation did not reveal any publication bias (Kendall’s Tau with continuity correction) (*p* = 0.79). Correction of the asymmetries via Duval and Tweedie’s “trim and fill” method yielded potentially missing studies for HR [1 missing study on the right side of the funnel plot, adjusted effect size=−5.94 bpm (95% CI: −8.59; −3.29)] (see funnel plot HR and publication bias in Supplementary Materials).

The funnel plots of standard error by effect size (SMD) were asymmetric (Figure 7B), suggesting potential publication biases in the meta-analysis of the hydration effect on the HRV index. The existence of publication biases was not confirmed by Egger’s linear regression (*p* = 0.12). Begg’s rank correlation did not reveal any publication bias (Kendall’s Tau with continuity correction (*p* = 0.14). Correction of the asymmetries via Duval & Tweedie’s “trim and fill” method yielded potentially missing studies for HRV [2 missing studies on the left side of the funnel plot, adjusted effect size = 0.54 (95% CI: 0.35, 0.72)] (see funnel plot HRV and publication bias in Supplementary Materials). With regards to the outcomes of SBP and DBP, the funnel chart was not applied because of the small number of included studies, as stated in the Cochrane Handbook [58].

## 4. Discussion

This systematic review with meta-analysis and meta-regression was finalized with the purpose of clarifying the effects of hydration on BP, HR and HRV index during and following the exercise period. As key findings, we observed that: (1) fluid intake lessened HR increases in response to physical exercise; (2) HRV recovery after exercise was improved in the fluid ingestion group; and (3) fluid intake demonstrated a modest increase in SBP compared to the control group following exercise. Our study is groundbreaking as a consequence of the consideration of both the effects of water and isotonic drink in different strategies of ingestion during exercise and recovery. In order to investigate if the difference in hydration strategies affects the results, we split the studies into subgroups.

In the HRV recovery outcome, we divided measurements into “water intake before and during exercise”, “water intake during exercise”, “water intake after exercise”, “isotonic intake during exercise” and “isotonic intake after exercise” in the recovery analysis that included, specifically, “water intake during exercise”, “water intake after exercise” and “isotonic intake after exercise” subgroups that reported acceleration of vagal recovery in the hydrated groups. When examining the “water intake before and during exercise” and “isotonic intake during exercise” subgroups, there were significant deviations. The hydration groups exhibited a small effect size (0.48) on HRV indexes. These results specify that water and isotonic intake before, during and after exercise were capable of accelerating post-exercise cardiac vagal recovery. We undertook a sensitivity analysis to assess the influence of each study on the final result. In this analysis, the withdrawal of specific studies did not affect the total results of the meta-analysis (see sensitivity analysis in Supplementary Materials).

Despite hydration status, these cardiovascular autonomic responses are conceivable since, at the close of the exercise session, inputs from the central nervous system are attenuated and the action of skeletal muscle mechanoreceptors ceases and induces the reactivation of the parasympathetic nervous system [15,64]. The additional phenomenon successions that explain vagal re-entry is the removal of muscle contraction product metabolites, such as hydrogen and lactate reduction of circulating catecholamines, blood pH and temperature returning to baseline values [15,65]. After these factors, there is a decrease in the activity of muscle metaboreceptors, chemoreceptors and thermoreceptors [15,66,67], creating a steady decline in HR mediated by vagal reentry and sympathetic withdrawal [15]. Then again, dehydration increases body temperature and, together with plasma hyperosmolarity, lessens vagal modulation and sustains high sympathetic modulation after exercise, which results in delayed cardiac autonomic recovery [36,68]. Charkoudian et al. [68] and Yun et al. [69] have suggested that hydration encourages the reduction of sympathetic activity as a side effect of enhanced vagal afferent activity in response to baroreceptor modulation during gastric distention. So, hydration becomes a simple and effective strategy to advance autonomic responses after exercise, which should be enforced to improve thermoregulation and lessen the cardiovascular effort in states facilitated by exercise [70].

In order to compensate for plasma volume reduction due to sweating during exercise, HR is increased to maintain cardiac output and, therefore, blood flow to attain the metabolic requirements of active muscles [57]. The results of our meta-analysis and meta-regression support this phenomenon. HR outcomes were divided into “water intake before and during exercise”, “water intake only before exercise”, “water intake during exercise” and “isotonic intake during exercise”. The analysis, including all subgroups, indicated a beneficial effect of hydration on HR during exercise, such as lower HR values compared to the control group.

In the “water intake before and during exercise” and “water intake during exercise” subgroups, we recognized reduced HR increase during exercise in comparison to the control group. Yet, in the “water intake only before exercise” (*p* = 0.06) subgroups, we did not note significant differences compared to the control group. Despite this, the decrease in HR under the effect of hydration compared to the control protocol persisted.

The fluid ingestion group sustained a mean HR = −6.20 bpm lower than the control group during exercise. The sub-groups “water intake before and during exercise” −6.20 bpm (95% CI: −11.70, −0.71) and “isotonic intake during exercise” −7.23 bpm (95% CI: −11.68, −3.71) presented lower HR in contrast to the control group. Regarding this, we proposed that water intake before and during exercise avoids greater increases in HR during exercise and could be used as a hydration strategy, especially when a rehabilitation program is anticipated [71]. Similarly, when hydrated before exercise, individuals seem to respond better to fluid loss during training, maximizing performance and safety in the training session [40], notwithstanding the fluid ingestion is critical to keep HR diminished.

Under this scenario, it has been established that HR changes during exercise are a reliable predictor of sudden death [72]. Cole et al. [64] discovered the necessity of HR recovery following exercise (as a reflection of parasympathetic modulation return) to predict mortality. In both studies, when deviations in HR were greater during exercise or delayed to lower values after exercise, the participants similarly gave an increased number of sudden deaths and overall mortality during the follow-up period.

In our study, meta-regression analysis was completed to evaluate the association between changes in HR with” body weight loss”. Our results uncovered a positive association between HR-lowering hydration and the “body weight loss” covariate (two-sided *p* value = 0.01). Our data highlighted that elevated weight loss potentially caused by fluid loss during exercise evokes higher values in HR, particularly when weight loss reaches values greater than 2% body weight, which represents significant dehydration [6]. Together the covariates “body weight loss”, “exercise time” and “temperature” were likewise responsible for 73% of the heterogeneity of our results (R^2^ analog = 0.73). Other covariables were studied; “BMI”, “mean age”, “exercise intensity” and “allocation” were observed, and no association with HR was detected (see “meta-regression data 2” in Supplementary Materials).

During the sensitivity analyses, we emphasized one study’s observations in the clinical measurement subgroup of HR. The study conducted by Lynn et al. [56] overestimates the positive results of hydration when compared to the others. Hence, in that analysis, we removed both groups to comprehend if, without their influence, the positive results would be maintained. When we oversee the study by Lynn et al. [56] as a group, we noticed a small decrease in the hydration effect that went from −6.20 bpm (−8.69, −3.71) to −5.99 bpm (−8.64, −3.35). In Lynn et al. [56] group B, we observed a modest reduction in the hydration effect to −5.56 bpm (−7.42, −3.70). We tested the reliability of the results, and we opted for a conservative analysis removing both studies at the same time from the statistics. In this situation, HR values decreased to −5.19 bpm (−7.00, −3.37) (see Sensitivity Analysis in Supplementary Materials). The omission of studies was unable to modify the final result of the meta-analysis. Consequently, the sensitivity analysis verified the robustness and reliability of these results for clinical application. We similarly performed a sensitivity analysis to attempt to explain some of the heterogeneity present in the statistics. Additionally, when we omitted both of these groups, the heterogeneity had a substantial decrease (I^2^ from 75% to 38%). Sensitivity analysis was attained in all studies of HR outcomes (see Sensitivity Analysis in Supplementary Materials).

BP measured after exercise presents lower values in the control protocol, notwithstanding the increase of −2 mmHg suggesting a spurious effect and not a clinically relevant effect. Analyzing BP by subgroups, they were divided into the “water intake before exercise”, “water intake during exercise”, “water intake after exercise” and “isotonic intake after exercise” subgroups. Only the “water intake after exercise” subgroup analysis for SBP (*p* = 0.04) recognized significant differences between hydration and control intervention. The meta-analysis confirmed that hydration was *slightly* higher than the control on post-exercise SBP values.

This BP behavior is reinforced by the scientific research literature since, after water intake, gastric distension stimulates mechanoreceptors in the stomach, causing sympathetic activation and increasing peripheral resistance [30,73]. One more phenomenon that explains the increase in BP in the hydration group is the mechanism of heat dissipation during exercise. The function of a mechanism such as this is capable of reducing plasma volume. The increase in sweating and water transfer from the intravascular to the interstitial medium decreases water production and inevitably stroke volume, with fluid replacement activated by hydration [37,55].

Yet, while this result is upheld by the scientific research literature, we must interpret it cautiously. Our sensitivity analysis exposes the fragility of this explanation. When we removed the study conducted by Vanderlei et al. [27] (*p* = 0.07) or Paula-Ribeiro et al. [52] (*p* = 0.17) or Teixeira et al. [31] (*p* = 0.13) or Vanderlei et al. [52] (*p* = 0.07), the result of SBP recovery is no more significant. In the DBP meta-analysis, we attained significant differences only when we removed the study by conducting Teixeira et al. [31] (on control, *p* < 0.001). We emphasized a study conducted by Paula-Ribeiro et al. [30] that overestimates the results in the control protocol and is accountable for the whole heterogeneity of statistics (I^2^ = 56%). The cited study might be an outlier.

## 5. Strength and Limitations

The characteristics identified in this meta-analysis will offer some guiding principles for upcoming studies to enhance the understanding of the effects of hydration on the cardiovascular health of individuals during a session of exercise. Many studies were not randomized as they enforced the control protocol as a control to establish the hydration strategy. To begin with, this proved to be problematic, yet our meta-regression illustrated that there was no statistical difference between randomized and non-randomized studies (see meta-regression data 2 “allocation” in Supplementary Materials). Since the number of studies on isotonic intake and cardiovascular parameters is still restricted, further research is expected to provide guidance concerning this intervention. We recommend further studies to better investigate BP recovery at least 30 minutes after exercise to evaluate the post-exercise hypotension and offer a more robust analysis and future guidance. We should not extrapolate our results for wheelchair athletes, women with pure autonomic failure, males with coronary artery disease, males with arterial hypertension and athletes with sickle cell owing to a limited number of studies. Our systematic review addressed the general query of whether hydration is able to influence cardiovascular parameters. We advocate more primary studies and, where possible, a specific systematic review of those populations.

This systematic review endorses that water ingestion before and during exercise or just during exercise, but not only before exercise reduces HR increase induced by physical exercise; the cardiac vagal modulation is higher in the fluid ingestion group, signifying an improvement in cardiac autonomic control restoration after exercise. A slight increase following exercise was observed in systolic blood pressure but without changes in DBP compared to the control group.

## 6. Conclusions

Our results suggest that hydration-attenuated exercise-induced increases in HR during exercise improved autonomic recovery through the quickening of cardiac vagal modulation in response to exercise, and causes a modest increase in SBP values. Yet, it lacks effects on DBP after exercise.

## Figures and Tables

**Table 1:** Description of the characteristics of the study population of articles by author and year, sample, age (years), mass (kg), height (cm), body fat (%), (mean ± SD or, [min–max]), exercise protocol and average peak oxygen (ml/kg/min).

Author/ Years/Country	Study Design	Sample	Age (Years)	Mass (kg)	Height (cm)	Body Fat (%)	Exercise Protocol/Environmental Temperature/Interval between Protocols	Average Peak Oxygen (ml/kg/min)	Hydration	Control
Berkulo et al. (2015) [22] South Africa	Non-RCT (Crossover)	12 trained male cyclists	34.3 ± 7	80.4 ± 6.4	183 ± 7	14.3 ± 3.6	Time trial (40 km)/35.2 ± 0.2 °C/ at least a minimum of a 48 h interval	53.5 ± 4.4	Water ingestion during exercise (12 mL/kg^−1^): divided into six equal portions every a 10 min	Non-fluid ingestion
Zacharakis et al. (2013) [23] Greece	RCT (Parallel)	8 SCI well-conditioned wheel 9 college students	SCI: 31.4 ± 8.4 PES: 20.1 ± 0.8	SCI-F: 72.8 ± 8.5 SCI-NF: 72.1 ± 11.5 PES-F: 74.1 ± 5.4 PES-NF: 73.9 ± 5.5	SCI: 180 ± 7 PES: 179 ± 5	Not reported	Wheelchair prolonged exercise (60 min at 55% VO_2_Max)/not reported/at least a minimum of a 5–7 days interval	SCI: 23.46 ± 1.38 PES: 35.13 ± 1.35	Water ingestion during exercise (did not report amount), equivalent to ~85% sweat loss, previously tested	Non-fluid ingestion
Hasegawa et al. (2006) [24] Japan	Non-RCT (Crossover)	9 untrained males	21.8 ± 0.8	61.7 ± 2.1	172 ± 20	15.1 ± 1.1	Cycle ergometer (60 min at 60% VO_2_Max)/39.1 ± 0.18 °C/at least a minimum of a 96 h interval	48.5 ± 1.5	Water ingestion during exercise (~0.85 L) divided into equal portions every 5 min, equivalent sweat loss	Non-fluid ingestion
McDermott et al. (2013) [25] USA	RCT (Crossover)	12 trained males	23.0 ± 4.0	81.3 ± 3.7	180 ± 6	7.8 ± 3.0	Walking and cycling alternated every 30 min (120 min at 40–60% VO_2_Max)/35.5 ± 1.5 °C/at least a minimum of a 5–7 days interval	56.9 ± 4.4	Water ingestion (~2 L) after exercise equivalent body weight loss	Non-fluid ingestion
Humm et al. (2008) [26] United Kingdom	Non-RCT (Crossover)	8 patients (5 women) with autonomic pure failure	63.9 ± 6.1	Not reported	Not reported	Not reported	Cycle ergometer in supine position (incremental test—25, 50 and 75 W each 3 min)/not reported/tests on the same day	Not reported	Distilled water ingestion (~0.48 L) immediately after exercise	Non-fluid ingestion
McConnell et al. (1999) [27] Australia		8 well-trained males	26.0 ± 1.0	79.6 ± 3.5	183 ± 2	Not reported	Cycling exercise (45 min at 80% VO_2_Max)/20.9 ± 0.2 °C/at least a minimum of a 96 h interval	63.4 ± 2.13	50% of the loss of body mass (~0.72 L water ingestion after exercise) 100% of the loss of body mass (~1.74 L water ingestion after exercise)	Non-fluid ingestion
Macartney et al. (2019) [28] Canada	Non-RCT (Crossover)	11 healthy males	26.0 ± 5.0	77.9 ± 8.3	181 ± 4	11.0 ± 4.0	Cycling exercise (90 min at 46%VO_2_Max/not reported/at least a minimum of a 72 h interval	60.5 ± 8.4	Water ingestion (0.5–0.7 L) after exercise, equivalent body weight loss previously tested	Non-fluid ingestion
Wingo et al. (2004) [29] Georgia	RCT (Crossover)	12 male mountain bikers	24.5 ± 1.1	76.9 ± 1.9	179 ± 2	14.3 ± 1.0	Mountain bike race (48 km)/30.8–31.18 °C/at least a minimum of a 5–7 days interval	Not reported	Water ingestion (~2.2 L) during exercise	Non-fluid ingestion
Paula-Ribeiro et al. (2013) [30] Brazil	RCT (Crossover)	14 healthy males	22.0 ± 1.4	74.0 ± 6.8	174 ± 1	Not reported	Cycle ergometer (30 min at 80% of respiratory compensation point)/18–24 °C/at least a minimum of a 48 h interval	46.7 ± 7.8	Water ingestion (7.5 ml/kg^−1^) during exercise	Non-fluid ingestion
Teixeira et al. (2014) [31] Brazil	RCT (Crossover)	13 healthy males	26.5 ± 5.9	76.3 ± 8.2	180 ± 1	Not reported	Bench press (3 sets of maximum repetition (RM) at 80% 1RM)/not reported/at least a minimum of a 48 h interval	Not reported	Water ingestion (0.5 L) immediately after exercise	Water ingestion (50 mL) immediately after exercise
Vianna et al. (2008) [32] Brazil	RCT (Crossover)	10 healthy subjects (8 men)	27.0 ± 3.0	68.7 ± 8.8	173 ± 7.6	Not reported	Cycling exercise (30 min at 80% anaerobic threshold)/not reported/at least a minimum of a 48 h interval	49.0 ± 8.4	Water ingestion (0.5 L) immediately after exercise	Water ingestion (50 mL) immediately after exercise
Laurino et al. (2021) [33] Brazil	Non-RCT (Crossover)	28 patient’s males with coronary artery disease (CAD)	63.6 ± 8.4	80.4 ± 12.9	171 ± 5	Not reported	Treadmill (40 min at 60–80% anaerobic threshold)/22–25 °C/at least a minimum of a 48 h interval	26.1 ± 5.27	Water ingestion (~0.34 L) during exercise every 10 min, equivalent to body weight loss previously tested	Non-fluid ingestion
Castro-Sepúldeva et al. (2015) [34] Chile	RCT (Parallel)	14 college male athletes	Rehydration group: 21.6 ± 1.2 Dehydration group: 20.7 ± 2.0	Rehydration group: 72.7 ± 2.5 Dehydration group: 75.1 ± 3.3	Rehydration group: 172 ± 1.5 Dehydration group: 175 ± 1.6	Not reported	Squats, push-ups, box jumps, 20 min sprints and slalom 20 m runs (45 min)/32 °C/at least a minimum of a 5–7 days interval	Not reported	Water ingestion (total amount not reported) equivalent to 150% of the body weight loss previously tested	Non-fluid ingestion
Armstrong et al. (1997) [35] USA	RCT (Crossover)	10 healthy males	21.0 ± 1.0	72.7 ± 2.13	174 ± 2.1	Not reported	Cycle ergometer (90 min at 47 ± 2% VO2max)/33 °C/at least a minimum of a 72 h interval	57.1 ± 1.5	Water ingestion (~1.65 L) during exercise	Non-fluid ingestion
Vanderlei et al. (2015) [36] Brazil	Non-RCT (Crossover)	31 healthy males	21.6 ± 1.8	72.6 ± 11.5	180 ± 1	Not reported	Treadmill (90 min at 60% VO_2_Max)/26.0 ± 2.34 °C/at least a minimum of a 48 h interval	46.8 ± 8.26	Water or isotonic ingestion every 15 min (total amount not reported) divided at 10 equal portions during exercise and recovery period equivalent to body weight loss previously tested	Non-fluid ingestion
Moreno et al. (2013) [37] Brazil	Non-RCT (Crossover)	31 healthy males	21.5 + 1.9	72.6 ± 11.5	170 ± 1	Not reported	Treadmill (90 min at 60% VO_2_Max)/26.0 ± 2.3 °C/at least a minimum of a 48 h interval	45.45 ± 8.26	Isotonic ingestion every 15 min (total amount not reported) divided at 10 equal portions during exercise and recovery period equivalent to body weight loss previously tested	Non-fluid ingestion
Heaps et al. (1994) [38] USA	Non-RCT (Crossover)	9 endurance-trained subjects (1 woman)	24 ± 6	77.7 ± 12.9	179 ± 15	Not reported	Cycle ergometer (20 min at 65% VO_2_Max)/different temperatures: 21 °C; 32 °C/not reported	52.76 ± 27.02	Water ingestion (total amount not reported) after exercise equivalent to body weight loss (~2.5% of body mass	Non-fluid ingestion
Severevyn et al. (2016) [39] Venezuela	Non-RCT (Crossover)	17 healthy male athletes	22.6 ± 2.1	Not reported	Not reported	Not reported	Stationary bike (30 min)/not reported/tests on the same day	Not reported	Water ingestion (total amount not reported) *ad libitum* after exercise	Non-fluid ingestion
Peçanha et al. (2014) [40] Brazil	RCT (Crossover)	12 healthy recreationally trained males	22.0 ± 1	74.0 ± 6.8	174 ± 1	Not reported	Cycling (60 rpm fixed, 80 ± 5% of % HRmax during 30 min)/18–24 °C/at least a minimum of a 48 h interval	43.24 ± 10.94	Water ingestion (7.5 ml/kg.^−1^) after exercise	Non-fluid ingestion
Endo et al. (2012) [41] Japan	Non-RCT (Crossover)	10 healthy males	(20–31)	(50–64)	(163–177)	Not reported	Cycling (60 min at 60% of HR reserve)/23 ± 0.5 °C/at least a minimum of a 48 h interval	Not reported	Water ingestion (~0.65 L) during exercise, equivalent to body weight loss previously tested	Non-fluid ingestion
Hamilton et al. (1991) [42] USA	Non-RCT (Crossover)	10 endurance-trained cyclists	27.8 ± 5.0	72.5 ± 5.0	Not reported	Not reported	Stationary bike (120 min at 70% VO_2_Max)/32 °C/at least a minimum of a 5–7 days interval	62.34 ± 4.82	Water ingestion (~2.34 L) after exercise, equivalent to body weight loss (previously tested)	Non-fluid ingestion
Mendonca et al. (2013) [43] USA	RCT (Crossover)	19 healthy adults (9 women)	20.9 ± 1.8	67.3 ± 10	172 ± 10	Not reported	Cycle ergometer ramp protocol (incremental test—increasing 25 W each 1 min)/not reported	45.2 ± 8.05	Water ingestion (0.5 mL) before exercise	Water ingestion (50 mL) before exercise
Ribeiro et al. (2004) [44] Brazil	RCT (Crossover)	8 males with arterial hypertension (AH) 8 healthy males (Control)	AH: 46.0 ± 3.0 Control: 48.0 ± 1	AH: 78.8 ± 2.5 Control: 79.5 ± 2.8	AH: 171 ± 2 Control: 167 ± 1	Not reported	Cycle ergometer (60 min at 40% VO_2_Peak)/18- 24 °C/at least a minimum of a 5–7 days interval	AH: 28.5 ± 1.5 Control: 29.9 ± 2.2	Water ingestion (460 mL) during exercise divided into 4 equal portions	Non-fluid ingestion
Melo-Marins et al. (2018) [45] Brazil	RCT (Crossover)	11 male recreational cyclists	30.0 ± 7.0	74.7 ± 10.6	177 ± 1	11.7 ± 0.5	Cycle ergometer (45 min at 70% of the maximal workload achieved in incremental test)/34 °C/at least a minimum of a 96 h interval	Not reported	Ad libitum protocol (~0.11 L water ingestion) Personalized volume (~1.08 L) water ingestion equivalent to sweat loss, previously tested	Non-fluid ingestion
Backhouse et al. (2007) [46] United Kingdom	Non-RCT (Crossover)	15 healthy males	21 ± 0.5	69.5 ± 1.4	Not reported	Not reported	Treadmill runs (90 min at 70% VO_2_Max)/19.7 ± 0.3 °C/at least a minimum of a 5–7 days interval	65.0 ± 1.2	Water ingestion (5 mL/kg.^−1^) before exercise, and (2 mL/kg.^−1^) every 20 min during exercise	Non-fluid ingestion
Lopez et al. (2011) [47] USA	RCT (Crossover)	14 endurance runners (7 women)	30.0 ± 10	66.7 ± 11.8	173 ± 7	14.3 ± 6.6	Time trial (12 km)/different temperatures: 27.6 ± 1.3 °C; 27.8 ± 1.6 °C; 26.3 ± 1.1 °C/at least a minimum of a 48 h interval	Not reported	Water ingestion (1.2 L) divided into equal 3 portions ingested every 4 km	Non-fluid ingestion
Tripette et al. (2010) [48] Western Africa	RCT (Crossover)	11 athletes with sickle cell trait (SCT) 12 athletes with normal hemoglobin (Control)	SCT: 26.4 ± 2.0 Control: 25.3 ± 1.9	SCT: 65.1 ± 7.0 Control: 70.2 ± 6.6	SCT: 176 ± 7 Control: 180 ± 10	Not reported	Cycling submaximal exercise (40 min at 55% aerobic peak power)/25–28 °C/at least a minimum of a 72 h interval	Not reported	SCT: water ingestion (~0.7 L) during exercise Control: water ingestion (~0.6 L) during	Non-fluid ingestion
Sanders et al. (1999) [49] South Africa	RCT (Crossover)	6 male cyclists	24.0 ± 2.0	78.0 ± 2.0	180 ± 1	Not reported	Cycling exercise (90 min at 65% VO_2_Peak)/32 °C/not reported	58.97 ± 2.56	Water ingestion (1.3 L) divided at 400 mL before starting exercise, and 100 mL every 10 min during exercise	Non-fluid ingestion
Schoffstal et al. (2001) [50] USA	RCT (Crossover)	10 experienced male competitive powerlifters	25.0 ± 1.0	85.5 ± 5.2	173.5 ± 1.7	17.8 ± 2.2	Bench press (5 to 7 attempts to 1RM)/not reported/at least a minimum of a 5–7 days interval	Not reported	Water ingestion (1.3 L) following exercise	Non-fluid ingestion
Silva et al. (2022) [51] Brazil	Non-RCT (Crossover)	30 male patients with CAD	63.7 ± 8.4	81.2 ± 12.9	171 ± 5	Not reported	Treadmill (40 min at 60–80% anaerobic threshold)/23–25 °C/at least a minimum of a 48 h interval	25.89 ± 5.26	Water ingestion (~0.34 L) during exercise every 10 min, equivalent to body weight loss previously tested	Non-fluid ingestion
Moreno et al. (2012) [52] Brazil	Non-RCT (Crossover)	31 healthy physically active males	21.5 ± 1.8	72.6 ± 11.5	177 ± 8	Not reported	Treadmill (90 min at 60% VO_2_Peak)/26 ± 2.34 °C/at least a minimum of a 48 h interval	46.40 ± 8.26	Isotonic ingestion every 15 min (total amount not reported) divided into 10 equal portions during exercise and recovery period equivalent to body weight loss previously tested	Non-fluid ingestion
de Oliveira et al. (2011) [53] Brazil		10 physically active subjects (3 women)	23.6 ± 4.0	Not reported	Not reported	Not reported	Cycle ergometer (20 min at 75 W for women, and 100 W for men)/not reported	Not reported	Water ingestion (500 mL) immediately after exercise	Non-fluid ingestion
Moreno et al. (2013) [54] (II) Brazil	Non-RCT (Crossover)	31 healthy physically active males	21.5 ± 1.8	72.6 ± 11.5	177 ± 8	Not reported	Treadmill (90 min at 60% VO_2_Peak)/26 ± 2.3 °C/at least a minimum of a 48 h interval	46.40 ± 8.26	Water ingestion every 15 min (total amount not reported) divided into 10 equal portions during exercise and recovery period equivalent to body weight loss previously tested	Non-fluid ingestion
Vanderlei et al. (2013) [55] Brazil	Non-RCT (Crossover)	31 healthy physically active males	21.5 ± 1.8	72.6 ± 11.5	177 ± 8	Not reported	Treadmill (90 min at 60% VO_2_Peak)/26 ± 2.34 °C/at least a minimum of a 48 h interval	46.40 ± 8.26	Water or isotonic ingestion every 15 min (total amount not reported) divided into 10 equal portions during exercise and recovery period equivalent to body weight loss previously tested	Non-fluid ingestion
Lynn et al. (2009) [56] USA	RCT (Crossover)	14 endurance- trained male athletes	26.5 ± 6.9	73.5 ± 9.9	179 ± 9	Not reported	Cycling exercise (60 min at 60% VO_2_Peak)/different temperatures: 22 °C; 30 °C/at least a minimum of a 72 h interval	63.40 ± 6.2	Water ingestion (total amount not reported) during exercise was equivalent to body weight loss previously tested	Non-fluid ingestion
Montain and Coyle et al. (1992) [57] USA	RCT (Crossover)	8 endurance-trained cyclists	23 ± 3	NF 72.22 ± 3.89 SF 71.87 ± 3.87 MF 71.54 ± 3.78 LF 71.88 ± 3.85	Not reported	Not reported	2 h of cycling exercise (62% of VO2 max)/32.7 ± 0.2 °C/at least a minimum of a 72 h interval	Not reported	Small (SF), moderate (MF) and large (LF) fluid volumes needed to replace ~20, 50, and 80% (respectively) of sweat loss during exercise Divided into 7 portions intake before and during exercise During LF, the total volume of fluid to be ingested was also divided into seven aliquots, but the first two drinks consumed each contained 20% of the total volume	Non-fluid ingestion

**Table 2:** GRADE: summary of findings.

**Fluid replacement vs. non-fluid replacement protocols (before, during and after exercise) repercussions on HR, HRV and BP.**					
**Patient or population:** athletes, physically active subjects and patients with cardiac disease. **Intervention:** fluid replacement (water and isotonic ingestion). **Comparison:** non-fluid replacement (no liquid ingestion or water intake up to 50 mL).					
**Outcome** **№ of Participants** **(Studies)**	**Anticipated absolute effects (95% CI)**			**Certainty**	**What Happens**
	**Comparison**		**Intervention** **(Difference)**		
**Fluid vs. non-fluid ingestion before, during or following exercise on HR values during exercise** № of participants: 820 (24 studies)	The mean HR was **141** (bpm)	-	MD −**5.94 bpm lower** (8.59 lower to 3.29 lower)	⨁⨁⨁⨁ **HIGH** Due to serious risk of bias. Upgraded due to large magnitude of effect. Upgraded because all plausible confounding would suggest a spurious effect.	Hydration protocol before and during exercise results in a less increase in the values of HR.
***Sub-group*: water intake before and during exercise on HR values during exercise** № of participants: 96 (4 studies)	The mean HR was **152** (bpm)	-	MD −**6.20 bpm lower** (11.70 lower to 0.71 lower)	⨁⨁⨁◯ **MODERATE** Due to serious risk of bias. Due to serious imprecision. Upgraded because all plausible confounding would suggest a spurious effect.	Water ingestion before and during exercise may result in a less increase in the values of HR.
***Sub-group*: water intake only before exercise on HR values during exercise** № of participants: 69 (4 studies)	The mean HR was **131** (bpm)	-	MD −**4.60 bpm lower** (9.41 lower to 0.22 higher)	⨁⨁◯ ◯ **LOW** Due to serious risk of bias. Due to serious inconsistency. Due to serious imprecision. Upgraded because all plausible confounding would suggest a spurious effect.	Water intake only during exercise showed a trend to avoid greater increases in HR during exercise, albeit these results were not significant.
***Sub-group*: water intake only during exercise on HR values during exercise** № of participants: 546 (15 studies)	The mean HR was **141** (bpm)	-	MD −**6.12 bpm lower** (9.35 lower to 2.89 lower)	⨁⨁⨁⨁ **HIGH** Due to serious risk of bias. Upgraded due to large magnitude of effect. Upgraded because all plausible confounding would suggest a spurious effect.	Water ingestion during exercise results in a less increase in the values of HR.
***Sub-group*: isotonic intake only during exercise on HR values during exercise** № of participants: 123 (3 studies)	The mean HR was **154.3** (bpm)	-	MD −**7.23 bpm lower** (11.68 lower to 2.79 lower)	⨁⨁◯◯ **LOW** Due to serious risk of bias. Upgraded due to large magnitude of effect. Upgraded because all plausible confounding would suggest a spurious effect.	Isotonic ingestion during exercise results in a less increase in the values of HR.
**Fluid vs. non-fluid ingestion before, during or following exercise** **on HRV values after exercise** № of participants: 96 (11 studies)	The mean HRV was **0.48** (SMD)	-	SMD **0.48 higher** (0.30 higher to 0.67 higher)	⨁⨁⨁◯ **MODERATE** Due to serious risk of bias. Due to serious imprecision. Upgraded because all plausible confounding would suggest a spurious effect.	Hydration protocol on recovery exercise results in a higher HRV following exercise.
**Fluid vs. non-fluid ingestion before, during or following exercise** on SBP values after exercise № of participants: 333 (7 studies)	The mean SBP was **122** mmHg	-	MD **2.55 mmHg higher** (0.08 higher to 4.42 higher)	⨁⨁◯ ◯ **LOW** Due to serious risk of bias. Due to serious inconsistency. Due to serious imprecision. Due to strongly suspected publication bias. Upgraded because all plausible confounding would suggest a spurious effect.	Fluid vs. non-fluid ingestion protocols present no significant difference in SBP values following exercise.
**Fluid vs. non-fluid ingestion before, during or following exercise** **on DBP values after exercise** № of participants: 333 (7 studies)	The mean DBP ambulatory 24 hrs was **68** mmHg	-	MD **3.02 mmHg higher** (−1.19 lower to 7.24 higher)	⨁⨁◯ ◯ **LOW** Due to serious risk of bias. Due to serious inconsistency. Due to serious imprecision. Due to strongly suspected publication bias. Upgraded because all plausible confounding would suggest a spurious effect.	Hydration protocol results in a higher DBP value following exercise.

**The risk in the intervention group** (and its 95% CI) is based on the assumed risk in the comparison group and the **relative effect** of the intervention (and its 95% CI). **CI:** confidence interval; **MD:** mean difference. The level of evidence was rated from Very low (⨁⨁◯ ◯) to High (⨁⨁⨁⨁)

## Data Availability

Data to support findings can be found in the supplementary material.

## Supplementary Materials

The following supporting information can be downloaded at: https://www.mdpi.com/article/10.3390/nu15214534/s1.
