# Supplementary material for: Influence of Fluid Ingestion on Heart Rate, Cardiac Autonomic Modulation and Blood Pressure in Response to Physical Exercise: A Systematic Review with Meta-Analysis and Meta-Regression
Source: Nutrients. 2023 Oct 26;15(21):4534. doi: 10.3390/nu15214534 (PMC10650885; doi:10.3390/nu15214534)
Supplement: Supplementary file 1 [file nutrients-15-04534-s001.zip › nutrients-2447126-supplementary.pdf]

Search strategy

	PubMed	EMBASE	Cochrane Library
**O<br>R**	("Heart Rate<br>Variability"<br>"Autonomic recovery"<br>"Vagal Reactivation"<br>"Heart rate recovery"<br>"Blood Pressure"<br>"Heart Rate”)	('Heart Rate Variability'<br>'Autonomic recovery'<br>'Vagal Reactivation'<br>"Heart rate recovery"<br>'Blood Pressure'<br>"Heart Rate')	Heart Rate Variability<br>Autonomic recovery<br>Vagal Reactivation<br>Heart rate recovery<br>Blood Pressure<br>Heart Rate
	**AND**	**AND**	**AND**
**O<br>R**	("Hydration"<br>"Fluid replacement"<br>"Water intake"<br>"Rehydration"<br>"Isotonic beverages"<br>“Sports beverages”)	('Hydration'<br>'Fluid replacement'<br>'Water intake'<br>'Rehydration'<br>'Isotonic beverages'<br>'Sports beverages')	Hydration<br>Fluid replacement<br>Water intake<br>Rehydration<br>Isotonic beverages<br>Sports beverages
**O<br>R**	("Exercise”<br>“Physical activity”)	('Exercise'<br>'Physical activity')	Exercise<br>Physical activity
	**CINAHL**	**LILACS ***	**WEB OF SCIENCE**
**O<br>R**	"Heart Rate Variability"<br>"Autonomic recovery"<br>"Vagal Reactivation"<br>"Heart rate recovery"<br>"Blood Pressure"<br>"Heart Rate	("Heart Rate<br>Variability"<br>"Autonomic recovery"<br>"Vagal Reactivation"<br>"Heart rate recovery"<br>"Blood Pressure"<br>"Heart Rate)	"Heart Rate Variability"<br>"Autonomic recovery"<br>"Vagal Reactivation"<br>"Heart rate recovery"<br>"Blood Pressure"<br>"Heart Rate"
	**AND**	**AND**	**AND**
**O<br>R**	"Hydration"<br>"Fluid replacement"<br>"Water intake"<br>"Rehydration"<br>"Isotonic beverages"<br>“Sports beverages”	("Hydration"<br>"Fluid replacement"<br>"Water intake"<br>"Rehydration"<br>"Isotonic beverages"<br>“Sports beverages”)	"Hydration"<br>"Fluid replacement"<br>"Water intake"<br>"Rehydration"<br>"Isotonic beverages"<br>“Sports beverages”
**O<br>R**	"Exercise”<br>“Physical activity”	("Exercise”<br>“Physical activity”)	"Exercise”<br>“Physical activity”

*the “Literatura Latino-Americana e do Caribe em Ciências da Saúde” also accepts acronym words in portuguese and spanigh. The strategy was executed in Portuguese and Spanish as well.

Pubmed Filter: Clinical Trial

EMBASE: Clinical Trial Controlled

Cochrane Library: Trial Section

Review authors' judgements about each risk of bias item for each included study

**Armstrong et. al 1997**		
**Bias**	**Authors' judgement**	**Support for judgement**
Random sequence generation (selection bias)	Unclear risk	“...the sequence of treatments was randomized and HST were separated by 3 days”<br>Comment: Insufficient detail reported
Allocation concealment(selection bias)	Unclear risk	“...and whether subjects consumed chilled water (,10–15°C) during exercise”<br>Comment: Insufficient detail reported
Blinding (performance bias)	High risk	Comment: Not possible to blind participants or researcher
Blinding of outcome assessors (detection bias) -	Unclear risk	Comment: Insufficient detail reported
Incomplete outcome data (attrition bias)	Low risk	Comment: No loss reported
Selective reporting (reporting bias)	Low risk	Comment: All pre-stated outcomes stated were reported
Other bias	Low risk	Comment: Appears to be free of other bias

**Berkulo et al. 2015**		
Bias	***Authors' judgement***	***Support for judgement***
Random sequence generation (selection bias)	High risk	“The order of experimental trials was counterbalanced among the participants with at least 48 hours between two tests”<br>Comment: Not reported
Allocation concealment(selection bias)	Unclear risk	“The total volume was divided in six equal portions to provide the participants water every 10 minutes”<br>Insufficient detail reported

Blinding (performance bias)	High risk	"(EU: sufficient fluid ingestion during the pre-exercise protocol to prevent any changes in BM + no fluid ingestion during the TT; HYPO: no fluid ingestion at all; FLUID: no fluid ingestion during the pre-exercise protocol + ad-libitum fluid ingestion during the TT..."<br>Comment: Not possible to blind participants or researcher
Blinding of outcome assessors (detection bias) -	Unclear risk	Comment: Insufficient detail reported
Incomplete outcome data (attrition bias)	Low risk	Comment: No loss reported
Selective reporting (reporting bias)	Low risk	Comment: All pre-stated outcomes stated were reported
Other bias	Low risk	Comment: Appears to be free of other bias

**Backhouse et. al 2007**		
**Bias**	**Authors' judgement**	**Support for judgement**
Random sequence generation (selection bias)	High risk	"Two trials were performed in a counterbalanced design, separated by an interval of at least 7 days"<br>Comment: Not reported
Allocation concealment(selection bias)	Unclear risk	"During the fluid replacement trial (FR-trial), a water bolus equivalent to 5.0 mL kg−1..."<br>Comment: Insufficient detail reported
Blinding (performance bias)	High risk	"No water was ingested during the no-fluid trial (NF-trial)."<br>Comment: Not possible to blind participants or researcher
Blinding of outcome assessors (detection bias) -	Unclear risk	Comment: Insufficient detail reported
Incomplete outcome data (attrition bias)	Low risk	Comment: No loss reported
Selective reporting (reporting bias)	Low risk	Comment: All pre-stated outcomes stated were reported
Other bias	Low risk	Comment: Appears to be free of other bias

**Castro-Sepúlveda et al. 2015**		
**Bias**	**Authors' judgement**	**Support for judgement**
Random sequence generation (selection bias)	Unclear risk	“Participants were cited to the laboratory at 10:00 am and randomly divided in two groups”<br>Comment: Insufficient detail reported
Allocation concealment(selection bias)	Unclear risk	“DG rested quietly during 4 h, while during the same time period participants from the RG receive a fluid intake”<br>Comment: Insufficient detail reported
Blinding (performance bias)	High risk	“DG rested quietly during 4 h, while during the same time period participants from the RG receive a fluid intake”<br>Comment: Not possible to blind participants
Blinding of outcome assessors (detection bias) -	Low risk	“T1 and T2 measurements were overseen by the same investigator, who was blinded to the hydration condition of participants”<br>Comment: Performed by a blinded researcher
Incomplete outcome data (attrition bias)	Low risk	“but because exclusion criteria (see below) only 14 were deemed suitable”<br>Comment: no loss reported after start of experiments
Selective reporting (reporting bias)	Low risk	“the purpose of this study is to investigate the effects of exercise-induced dehydration on HRV and RMR”<br>Comment: All pre-stated outcomes stated were reported
Other bias	Low risk	Comment: Appears to be free of other bias

**Endo et. al 2012**		
**Bias**	**Authors' judgement**	**Support for judgement**
Random sequence generation (selection bias)	High risk	“To perform the two study protocols, the subjects reported to the laboratory on two separate days at the same time”<br>Comment: not reported
Allocation concealment(selection bias)	Unclear risk	“during exercise: an oral water drinking protocol and a control (no water drinking) protocol.”<br>Comment: Insufficient detail reported
Blinding (performance bias)	High risk	“during exercise: an oral water drinking protocol and a control (no water drinking) protocol.”<br>Comment: Not possible to blind participants or researcher
Blinding of outcome assessors (detection bias) -	Unclear risk	Comment: Insufficient detail reported
Incomplete outcome data (attrition bias)	Low risk	Comment: All pre-stated outcomes stated were reported
Selective reporting (reporting bias)	High risk	“Post-exercise measurements were taken every 5 min for 1 h after cessation of exercise”<br>Comment: only 15 minute intervals were shown
Other bias	Low risk	Comment: Appears to be free of other bias

**Hamilton et. al 1991**		
**Bias**	**Authors' judgement**	**Support for judgement**
Random sequence generation (selection bias)	High risk	“This was performed on two occasions, in a balanced order, at l-wk intervals.”<br>Comment: Not reported
Allocation concealment(selection bias)	Unclear risk	“Drinking began within 5 min before exercise and continued at 2O-min intervals until 100 min”

		Comment: Insufficient detail reported
Blinding (performance bias)	High risk	"During one trial, no fluid was ingested during exercise (NF). During the fluid replacement (FR) trial, water was ingested at a rate that"<br>Comment: Not possible to blind participants or researcher
Blinding of outcome assessors (detection bias) -	Unclear risk	Comment: Insufficient detail reported
Incomplete outcome data (attrition bias)	Low risk	Comment: No loss reported
Selective reporting (reporting bias)	Low risk	Comment: All pre-stated outcomes stated were reported
Other bias	Low risk	Comment: Appears to be free of other bias

**Hasegawa et al. 2006**		
**Bias**	**Authors' judgement**	**Support for judgement**
Random sequence generation (selection bias)	High risk	Comment: Not reported
Allocation concealment(selection bias)	Unclear risk	"The total volume of water ingestion during the Water and Combined conditions was equal to the amount of fluid lost in the sweat test."<br>Comment: Insufficient detail reported
Blinding (performance bias)	High risk	" under four separate conditions: No Water intake, Precooling, Water ingestion at 5 min intervals, and a Combination of pre-cooling and water ingestion"<br>Comment: Not possible to blind participants or researcher
Blinding of outcome assessors (detection bias) -	Unclear risk	Comment: Insufficient detail reported
Incomplete outcome data (attrition bias)	Low risk	Comment: No loss reported
Selective reporting (reporting bias)	Low risk	Comment: All pre-stated outcomes stated were reported
Other bias	Low risk	Comment: Appears to be free of other bias

**Heaps et. al 1994**		
**Bias**	**Authors' judgement**	**Support for judgement**
Random sequence generation (selection bias)	High risk	Comment: Not reported
Allocation concealment(selection bias)	Unclear risk	Comment: Insufficient detail reported
Blinding (performance bias)	High risk	"On two separate occasions following exercise, subjects either received no fluid or ingested a volume of water equal to 100% of the fluid lost during exercise"<br>Comment: Not possible to blind participants or researcher
Blinding of outcome assessors (detection bias) -	Unclear risk	Comment: Insufficient detail reported
Incomplete outcome data (attrition bias)	Low risk	Comment: No loss reported
Selective reporting (reporting bias)	Low risk	Comment: All pre-stated outcomes stated were reported
Other bias	Low risk	Comment: Appears to be free of other bias

**Humm et al. 2008**		
**Bias**	**Authors' judgement**	**Support for judgement**
Random sequence generation (selection bias)	Unclear risk	"Patients were randomly assigned to start with the protocol without water drinking (termed protocol A; three patients) or with water intake (protocol B) and there were on average 10.8 days (SD 13.3) between the two assessments"
Allocation concealment(selection bias)	Unclear risk	"Patients were asked to drink the water within 5 min; the average time taken was 2 min 31 s"<br>Comment: Insufficient detail reported
Blinding (performance bias)	High risk	"The test protocol was performed without water ingestion and on a separate occasion after 480 ml of distilled water immediately after pre-exercise standing"

		Comment: Not possible to blind participants or researcher
Blinding of outcome assessors (detection bias) -	Unclear risk	Comment: Insufficient detail reported
Incomplete outcome data (attrition bias)	Low risk	Comment: No loss reported
Selective reporting (reporting bias)	Low risk	Comment: All pre-stated outcomes stated were reported
Other bias	Low risk	Comment: Appears to be free of other bias

**Laurino et. al 2021**		
**Bias**	**Authors' judgement**	**Support for judgement**
Random sequence generation (selection bias)	High risk	“study limitation, the randomization of the protocols was not possible because...”<br>Comment: Nonrandomized protocol
Allocation concealment(selection bias)	Unclear risk	“During the HP, volunteers ingested four equal portions of mineral water”<br>Comment: Insufficient detail reported
Blinding (performance bias)	High risk	“During the HP, volunteers ingested four equal portions of mineral water...”<br>Comment: Not possible to blind participants or researcher
Blinding of outcome assessors (detection bias) -	Low risk	“This analysis was performed by an experienced and blinded researcher.”<br>Comment: Performed by a blinded researcher
Incomplete outcome data (attrition bias)	Low risk	“In total, 31 subjects were recruited (Fig. 1). After sample losses, 28 subjects were analyzed”<br>Comment: Acceptable loss (9,6%)
Selective reporting (reporting bias)	Low risk	Comment: All pre-stated outcomes stated were reported
Other bias	Low risk	Comment: Appears to be free of other bias

**Lopez et al. 2011**		
**Bias**	**Authors' judgement**	**Support for judgement**
Random sequence generation (selection bias)	Unclear risk	"The study design consisted of a randomized, crossover,counterbalanced design" Comment: Insufficient detail reported
Allocation concealment(selection bias)	Unclear risk	"HY received 400 ml of water, whereas those in DHY received no fluids)" Comment: Insufficient detail reported
Blinding (performance bias)	High risk	"HY received 400 ml of water, whereas those in DHY received no fluids)." Comment: Not possible to blind participants or researcher
Blinding of outcome assessors (detection bias) -	Unclear risk	Comment: Insufficient detail reported
Incomplete outcome data (attrition bias)	Low risk	Comment: No loss reported
Selective reporting (reporting bias)	Low risk	Comment: All pre-stated outcomes stated were reported
Other bias	High risk	"...any woman who was pregnant; chronic health problems; a history of cardiovascular, metabolic, or respiratory disease; fever or current illness..." Comment: did not standardize the menstrual period of female volunteers.

**Lynn et al. 2009**		
**Bias**	**Authors' judgement**	**Support for judgement**
Random sequence generation (selection bias)	Unclear risk	"Each of the study days were randomized prior to the screening visit and order was divided and assigned to each subject" Comment: Insufficient detail reported

Allocation concealment(selection bias)	Unclear risk	"This amount of water was consumed during exercise for Fluid condition. No water was consumed during exercise for either Control or Warm condition."<br>Comment: Insufficient detail reported
Blinding (performance bias)	High risk	"This amount of water was consumed during exercise for Fluid condition. No water was consumed during exercise for either Control or Warm condition."<br>Comment: Not possible to blind participants or researcher
Blinding of outcome assessors (detection bias) -	Unclear risk	Comment: Insufficient detail reported
Incomplete outcome data (attrition bias)	High risk	"Control, Fluid, Warm (five subjects); Control, Warm, Fluid (four subjects); Warm, Control, Fluid (five subjects)."<br>Comment: 20% loss reported
Selective reporting (reporting bias)	Low risk	Comment: All pre-stated outcomes stated were reported
Other bias	Low risk	Comment: Appears to be free of other bias
**Macartney et. al 2019**		
**Bias**	**Authors' judgement**	**Support for judgement**
Random sequence generation (selection bias)	High risk	"effects of exercise in the heat and fluid loss, it was nonrandomized"<br>Comment: Nonrandomized protocol
Allocation concealment(selection bias)	Unclear risk	"Fluid (tap water equilibrated to chamber temperature ~40°C) was administered in boluses of 500 to 700 mL for 5 min before..."<br>Comment: Insufficient detail reported
Blinding (performance bias)	High risk	"In the first experimental session, participants performed the above-mentioned experimental

		protocol with no fluid replacement (No-FR; 3.4% reduction in body mass). The change in body weight measured in this session was then used to determine the rate of fluid replacement required to maintain body mass (and thereby euhydration) during the second or fluid replacement trial (FR)"<br>Comment: Not possible to blind participants or researcher
Blinding of outcome assessors (detection bias) -	Unclear risk	Comment: Insufficient detail reported
Incomplete outcome data (attrition bias)	Low risk	Comment: No loss reported
Selective reporting (reporting bias)	Low risk	Comment: All pre-stated outcomes stated were reported
Other bias	Low risk	Comment: Appears to be free of other bias

**McConell et al. 1999**		
**Bias**	**Authors' judgement**	**Support for judgement**
Random sequence generation (selection bias)	Unclear risk	"then the particular combination of trials was randomly assigned to each subject."<br>Comment: Insufficient detail reported
Allocation concealment(selection bias)	Unclear risk	"...During an experimental trial, subjects received either no fluid (NF) or fluid... "<br>Comment: Insufficient detail reported
Blinding (performance bias)	High risk	"...During an experimental trial, subjects received either no fluid (NF) or fluid... "<br>Comment: Not possible to blind participants or researcher
Blinding of outcome assessors (detection bias) -	Unclear risk	Comment: Insufficient detail reported
Incomplete outcome data (attrition bias)	Low risk	Comment: No loss reported
Selective reporting (reporting bias)	Low risk	Comment: All pre-stated outcomes stated were reported

Other bias	Low risk	Comment: Appears to be free of other bias

**McDermott et al. 2013**		
**Bias**	**Authors' judgement**	**Support for judgement**
Random sequence generation (selection bias)	Unclear risk	"The study followed a randomized, control comparison, crossover design"<br>Comment: Insufficient detail reported
Allocation concealment(selection bias)	Unclear risk	"After EXDE, the participants were helped to a seated position and were rehydrated"<br>Comment: Insufficient detail reported
Blinding (performance bias)	High risk	"ncluded no fluid (NF), ad libitum, oral (OR), intravenous (IV), and a combination of IV and OR (IV + OR) of 1/2-normal saline (0.45% NaCl)."<br>Comment: Not possible to blind participants or researcher
Blinding of outcome assessors (detection bias) -	Unclear risk	Comment: Insufficient detail reported
Incomplete outcome data (attrition bias)	Low risk	Comment: No loss reported
Selective reporting (reporting bias)	High risk	" whereas the HR was measured every 15 minutes during EXDE"<br>Comment: HR Outcome during exercise were not reported in results sections.
Other bias	Low risk	Comment: Appears to be free of other bias

**Melo-Marins et. al 2018**		
**Bias**	**Authors' judgement**	**Support for judgement**
Random sequence generation (selection bias)	Unclear risk	"The trials were randomized and interspersed by at least 1 week to..."<br>Comment: Insufficient detail reported

Allocation concealment(selection bias)	Unclear risk	“bottles containing 800 ml of water were available for participants to drink upon request”<br>Comment: Insufficient detail reported
Blinding (performance bias)	High risk	“In the control trial (CON), participants did not have access to drinks.”<br>Comment: Not possible to blind participants or researcher
Blinding of outcome assessors (detection bias) -	Unclear risk	Comment: Insufficient detail reported
Incomplete outcome data (attrition bias)	Low risk	Comment: No loss reported
Selective reporting (reporting bias)	Low risk	Comment: All pre-stated outcomes stated were reported
Other bias	Low risk	Comment: Appears to be free of other bias

**Mendonca et. al 2013**		
**Bias**	**Authors' judgement**	**Support for judgement**
Random sequence generation (selection bias)	Unclear risk	“randomized, counterbalanced manner...”<br>Comment: Insufficient detail reported
Allocation concealment(selection bias)	Unclear risk	“Subsequently, participants ingested either 50(control) or 500 ml (experimental) of water”<br>Comment: Insufficient detail reported
Blinding (performance bias)	Low risk	“protocols on two different occasions (after ingestion of 50and 500 ml of water)”<br>Comment: same intervation for both groups
Blinding of outcome assessors (detection bias) -	Unclear risk	Comment: Insufficient detail reported
Incomplete outcome data (attrition bias)	Low risk	Comment: No loss reported
Selective reporting (reporting bias)	Low risk	Comment: All pre-stated outcomes stated were reported
Other bias	High risk	Comment: HR data from men and women in the early follicular phase presented in the same group

**Montain et. al 1992**		
**Bias**	**Authors' judgement**	**Support for judgement**
Random sequence generation (selection bias)	Unclear risk	"During exercise, the subjects randomly received no fluid (NF) or ingested a small (SF), moderate (MF), or large (LF) volume of fluid, which replaced…"<br>Comment: Insufficient detail reported
Allocation concealment(selection bias)	Unclear risk	Comment: Insufficient detail reported
Blinding (performance bias)	High risk	"During exercise, the subjects randomly received no fluid (NF) or ingested a small (SF), moderate (MF), or large (LF) volume of fluid, which replaced "<br>Comment: Not possible to blind participants or researcher
Blinding of outcome assessors (detection bias) -	Unclear risk	Comment: Insufficient detail reported
Incomplete outcome data (attrition bias)	Low risk	Comment: No loss reported
Selective reporting (reporting bias)	Low risk	Comment: All pre-stated outcomes stated were reported
Other bias	Low risk	Comment: Appears to be free of other bias

**Moreno et. al 2012**		
**Bias**	**Authors' judgement**	**Support for judgement**
Random sequence generation (selection bias)	High risk	"In subsequent visits, called control (CP) and experimental (EP) protocols..."<br>Comment: Not reported
Allocation concealment(selection bias)	Unclear risk	"(Gatorade, Brazil) was administered in 10 equal portions at regular 15-minute intervals from the 15th minute of exercise..."<br>Comment: Insufficient detail reported
Blinding (performance bias)	High risk	"In addition, in PC there was no administration of isotonic drink, while in PE a hydroelectrolytic solution"

		Comment: Not possible to blind participants or researcher
Blinding of outcome assessors (detection bias) -	Unclear risk	Comment: Insufficient detail reported
Incomplete outcome data (attrition bias)	Low risk	“No volunteers were excluded during the course of the experimente”<br>Comment: No loss reported
Selective reporting (reporting bias)	Low risk	Comment: All pre-stated outcomes stated were reported
Other bias	Low risk	Comment: Appears to be free of other bias

**Moreno et. al 2013**		
**Bias**	**Authors' judgement**	**Support for judgement**
Random sequence generation (selection bias)	High risk	“In subsequent visits, called control (CP) and experimental (EP) protocols...”<br>Comment: Insufficient detail reported
Allocation concealment(selection bias)	Unclear risk	“The isotonic solution was administered in 10 equal portions at regular intervals of 15 min from the fifteenth minute of exercise until ...”<br>Comment: Insufficient detail reported
Blinding (performance bias)	High risk	“Volunteers were not given any fluids to drink during CP”<br>Comment: Not possible to blind participants or researcher
Blinding of outcome assessors (detection bias) -	Unclear risk	Comment: Insufficient detail reported
Incomplete outcome data (attrition bias)	Low risk	“No volunteers were excluded during the course of the experimente”<br>Comment: No loss reported
Selective reporting (reporting bias)	Low risk	Comment: All pre-stated outcomes stated were reported
Other bias	Low risk	Comment: Appears to be free of other bias

Moreno et. al 2013 (II)		
**Bias**	**Authors' judgement**	**Support for judgement**
Random sequence generation (selection bias)	High risk	"In subsequent visits, called control (CP) and experimental (EP) protocols..." Comment: Not reported
Allocation concealment(selection bias)	Unclear risk	"Water intake was administered in 10 equal portions at regular intervals of 15 min from the fifteenth minute of exercise until the end of the recovery...." Comment: Insufficient detail reported.
Blinding (performance bias)	High risk	"Volunteers were not given any fluids to drink during CP; however, they were given water..." Comment: Not possible to blind participants or researcher
Blinding of outcome assessors (detection bias) -	Unclear risk	Comment: Insufficient detail reported
Incomplete outcome data (attrition bias)	Low risk	"No volunteers were excluded during the course of the experimente" Comment: No loss reported
Selective reporting (reporting bias)	Low risk	Comment: All pre-stated outcomes stated were reported
Other bias	Low risk	Comment: Appears to be free of other bias

Paula-Ribeiro et. al 2013		
**Bias**	**Authors' judgement**	**Support for judgement**
Random sequence generation (selection bias)	Unclear risk	"The sessions were held randomly on 3 non-consecutive days in the afternoon" Comment: Insufficient detail reported

Allocation concealment(selection bias)	Unclear risk	“Immediately after the end of exercise, the subjects consumed 7.5 ml water per kg body weight (mean ingested volume of ~500 ml)”<br>Comment: Insufficient detail reported
Blinding (performance bias)	High risk	“The control session consisted of the same steps as the water intake session, except for postexercise water intake”<br>Comment: Not possible to blind participants or researcher
Blinding of outcome assessors (detection bias) -	Unclear risk	Comment: Insufficient detail reported
Incomplete outcome data (attrition bias)	Low risk	Comment: No loss reported
Selective reporting (reporting bias)	Low risk	Comment: All pre-stated outcomes stated were reported
Other bias	Low risk	Comment: Appears to be free of other bias

**Peçanha et al. 2011**		
**Bias**	**Authors' judgement**	**Support for judgement**
Random sequence generation (selection bias)	Unclear risk	“water intake or control session (randomized)...”<br>Comment: Insufficient detail reported
Allocation concealment(selection bias)	Unclear risk	Immediately after the end of the exercise, the subjects drank 500 ml of water at...”<br>Comment: Insufficient detail reported
Blinding (performance bias)	High risk	“in addition, a control session (CON), similar to the experimental one except for the absence of WI..”<br>Comment: Not possible to blind participants or researcher
Blinding of outcome assessors (detection bias) -	Unclear risk	Comment: Insufficient detail reported
Incomplete outcome data (attrition bias)	Low risk	Comment: No loss reported
Selective reporting (reporting bias)	Low risk	Comment: All pre-stated outcomes stated were reported

Other bias	Low risk	Comment: Appears to be free of other bias

**Peçanha et al. 2014**		
**Bias**	**Authors' judgement**	**Support for judgement**
Random sequence generation (selection bias)	Unclear risk	“The experimental sessions were randomly performed on two nonconsecutive days”<br>Comment: Insufficient detail reported
Allocation concealment(selection bias)	Unclear risk	“Immediately after exercise, within 30 second, the volunteers: a) ingested a volume of 7.5 ml of water per kg of body weight [WI session; mean volume ingested ~500 ml] or b) performed no water consumption“<br>Comment: Insufficient detail reported
Blinding (performance bias)	High risk	““Immediately after exercise, within 30 second, the volunteers: a) ingested a volume of 7.5 ml of water per kg of body weight [WI session; mean volume ingested ~500 ml] or b) performed no water consumption“<br>Comment: Not possible to blind participants or researcher
Blinding of outcome assessors (detection bias) -	Unclear risk	Comment: Insufficient detail reported
Incomplete outcome data (attrition bias)	Low risk	Comment: No loss reported
Selective reporting (reporting bias)	Low risk	Comment: All pre-stated outcomes stated were reported
Other bias	Low risk	Comment: Appears to be free of other bias

**Ribeiro et. al 2004**		
**Bias**	**Authors' judgement**	**Support for judgement**
Random sequence generation (selection bias)	Unclear risk	“the individuals exercised randomly on two occasions separated by a one-week interval: with and without pure water drinking (control) ”<br>Comment: Insufficient detail reported
Allocation concealment(selection bias)	Unclear risk	“During the water-exercise situation, the individuals ingested pure water”<br>Comment: Insufficient detail reported
Blinding (performance bias)	High risk	“The estimated total amount of water (460 ml) during exercise was divided into 4 equal doses ingested at 15-min intervals. Before and during the control-exercise situation the individuals did not receive any water.”<br>Comment: Not possible to blind participants or researcher
Blinding of outcome assessors (detection bias) -	Unclear risk	Comment: Insufficient detail reported
Incomplete outcome data (attrition bias)	Low risk	Comment: No loss reported during trials
Selective reporting (reporting bias)	Low risk	Comment: All pre-stated outcomes stated were reported
Other bias	Low risk	Comment: Appears to be free of other bias

**Sanders et al. 1999**		
**Bias**	**Authors' judgement**	**Support for judgement**
Random sequence generation (selection bias)	Unclear risk	“Each subject performed a random order of three experimental trials, each separated by 1 week...”<br>Comment: Insufficient detail reported
Allocation concealment(selection bias)	Unclear risk	“During the rides, the subjects either consumed no fluid or they ingested 400 ml of water.”<br>Comment: Insufficient detail reported

Blinding (performance bias)	High risk	“During the rides, the subjects either consumed no fluid or they ingested 400 ml of water.”<br>Comment: Not possible to blind participants or researcher
Blinding of outcome assessors (detection bias) -	Unclear risk	Comment: Insufficient detail reported
Incomplete outcome data (attrition bias)	Low risk	Comment: No loss reported
Selective reporting (reporting bias)	Low risk	Comment: All pre-stated outcomes stated were reported
Other bias	Low risk	Comment: Appears to be free of other bias

**Schoffstall et. al 2001**		
**Bias**	**Authors' judgement**	**Support for judgement**
Random sequence generation (selection bias)	Unclear risk	“Subjects were matched on baseline estimated 1RM and randomly assigned to 1 of 2 groups...”
Allocation concealment(selection bias)	Unclear risk	“, all subjects were given a standard time of 2 hours to rehydrate during which they consumed water ad libitum in order...”<br>Comment: Insufficient detail reported
Blinding (performance bias)	High risk	“dehydration (D, ;2 hours in a sauna) followed by rehydration (R, ;2 hours of rest with water ad libitum.”<br>Comment: Not possible to blind participants or researcher
Blinding of outcome assessors (detection bias) -	Unclear risk	Comment: Insufficient detail reported
Incomplete outcome data (attrition bias)	Low risk	Comment: No loss reported
Selective reporting (reporting bias)	Low risk	Comment: All pre-stated outcomes stated were reported
Other bias	Low risk	Comment: Appears to be free of other bias

**Severeyn et al. 2016**		
**Bias**	**Authors' judgement**	**Support for judgement**
Random sequence generation (selection bias)	High risk	Comment: Not reported
Allocation concealment(selection bias)	Unclear risk	"...acquisition, individuals were hydrated with water ad libitum..."<br>Comment: Insufficient detail reported
Blinding (performance bias)	High risk	Comment: Not possible to blind participants or researcher
Blinding of outcome assessors (detection bias) -	Unclear risk	Comment: Insufficient detail reported
Incomplete outcome data (attrition bias)	Low risk	Comment: No loss reported
Selective reporting (reporting bias)	Low risk	Comment: All pre-stated outcomes stated were reported
Other bias	High risk	"After the second electrocardiographic acquisition, individuals were hydrated with water ad libitum, and 30 minutes after hydration, they underwent a third electrocardiographic acquisition"<br>Comment: Based on the data presented, it is not known whether recovery is improved by hydration or is natural.

**Silva et. al 2022**		
**Bias**	**Authors' judgement**	**Support for judgement**
Random sequence generation (selection bias)	High risk	"The study is a cross-over clinical trial"<br>Comment: Not reported
Allocation concealment(selection bias)	Unclear risk	"The consumption was done in 8 equal portions, taken at regular intervals of 10 minutes at the exercise ..."<br>Comment: Insufficient detail reported
Blinding (performance bias)	High risk	"the participants performed the same CP activities, with mineral water (Bonafont, Brasil) intaker..."

		Comment: Not possible to blind participants or researcher
Blinding of outcome assessors (detection bias) -	Unclear risk	Comment: Insufficient detail reported
Incomplete outcome data (attrition bias)	Low risk	"In total, 31 subjects were recruited (Fig. 1). After sample losses, 29 subjects were analyzed"<br>Comment: Acceptable loss (6,4%)
Selective reporting (reporting bias)	Low risk	Comment: All pre-stated outcomes stated were reported
Other bias	Low risk	Comment: Appears to be free of other bias

**Teixeira et al. 2014**		
**Bias**	**Authors' judgement**	**Support for judgement**
Random sequence generation (selection bias)	Unclear risk	"...chosen randomly at each visit."<br>Comment: Insufficient detail reported
Allocation concealment(selection bias)	Unclear risk	"500 ml (mean ± SD6.6 ± 0.7 ml/kg; experimental visit) or 50 ml (control visit)<br>Comment: Insufficient detail reported
Blinding (performance bias)	Low risk	500 ml (mean ± SD6.6 ± 0.7 ml/kg; experimental visit) or 50 ml (control visit)<br>Comment: same intervation for both groups
Blinding of outcome assessors (detection bias) -	Unclear risk	Comment: Insufficient detail reported
Incomplete outcome data (attrition bias)	Low risk	"No volunteers were excluded during the execution of the current study"<br>Comment: No loss reported
Selective reporting (reporting bias)	Low risk	Comment: All pre-stated outcomes stated were reported
Other bias	Low risk	Comment: Appears to be free of other bias

**Tripette et. al 2010**		
**Bias**	**Authors' judgement**	**Support for judgement**
Random sequence generation (selection bias)	Unclear risk	“the same subjects participated in two randomized sessions (3–5 days apart)..”<br>Comment: Insufficient detail reported
Allocation concealment(selection bias)	Unclear risk	“In one session,subjects could not drink water (Dehyd), whereas in the other theycould drink water ad libitum (Hyd) during exercise.”<br>Comment: Insufficient detail reported
Blinding (performance bias)	High risk	“In one session,subjects could not drink water (Dehyd), whereas in the other theycould drink water ad libitum (Hyd) during exercise.”<br>Comment: Not possible to blind participants or researcher
Blinding of outcome assessors (detection bias) -	Unclear risk	Comment: Insufficient detail reported
Incomplete outcome data (attrition bias)	Low risk	Comment: No loss reported
Selective reporting (reporting bias)	High risk	“maximum HR, and the volume of water ingestedwere compared between the two groups using ...”<br>“HR and rectal temperature did not differ between the Hyd and Dehyd conditions at the end of exercise”<br>Comment: it is unclear which moment is "end of exercise". HRpeak not reported for HYD and Dhyd.
Other bias	Low risk	Comment: Appears to be free of other bias

**Vanderlei et. al 2013**		
**Bias**	**Authors' judgement**	**Support for judgement**
Random sequence generation (selection bias)	High risk	“In the subsequent steps the volunteers performed three different protocols, namely the control protocol

		(CP), the hydration with water protocol (PE1) and the hydration with isotonic drink protocol (PE2)..."<br>Comment: Not reported
Allocation concealment(selection bias)	Unclear risk	"the liquids were administered in 10 equal portions at regular 15- minute intervals, commencing 15 minutes a...."<br>Comment: Insufficient detail reported
Blinding (performance bias)	High risk	"In CP no hydrating solution was administered, whereas in PE1 water was given, and PE2 received a hydroelectrolytic solution..."<br>Comment: Not possible to blind participants or researcher
Blinding of outcome assessors (detection bias) -	Unclear risk	Comment: Insufficient detail reported
Incomplete outcome data (attrition bias)	Low risk	"No volunteers were excluded during the course of the experimente"<br>Comment: No loss reported
Selective reporting (reporting bias)	Low risk	Comment: All pre-stated outcomes stated were reported
Other bias	Low risk	Comment: Appears to be free of other bias

**Vanderlei et. al 2015**		
**Bias**	**Authors' judgement**	**Support for judgement**
Random sequence generation (selection bias)	High risk	"volunteers performed three different protocols, control protocol, protocol with water intake, and protocol with ingestion of isotonic solution"<br>Comment: Not reported
Allocation concealment(selection bias)	Unclear risk	"...Hydration was administered in 10 equal portions and at regular 15-min intervals from the 15th minute of exercise..."<br>Comment: Insufficient detail reported

Blinding (performance bias)	High risk	“...with ingestion of isotonic solution, which consisted of 10 min of rest in the supine position, followed by 90 min of exercise...”<br>“...Hydration was administered in 10 equal portions and at regular 15-min intervals from the 15th minute of exercise until the end of the recovery period...”<br><br>Comment: Not possible to blind participants or researcher
Blinding of outcome assessors (detection bias) -	Unclear risk	Comment: Insufficient detail reported
Incomplete outcome data (attrition bias)	Low risk	Comment: No loss reported
Selective reporting (reporting bias)	Low risk	Comment: All pre-stated outcomes stated were reported
Other bias	Low risk	Comment: Appears to be free of other bias

**Vianna et al. 2008**		
**Bias**	**Authors' judgement**	**Support for judgement**
Random sequence generation (selection bias)	Unclear risk	“Immediately after exercise subjects drank room temperature water within 90 s, either 500 ml (experimental) or 50 ml (control), chosen randomly at each visit”<br>Comment: Insufficient detail reported
Allocation concealment(selection bias)	Unclear risk	Immediately after exercise subjects drank room temperature water within 90 s, either 500 ml (experimental) or 50 ml (control).<br>Comment: Insufficient detail reported
Blinding (performance bias)	Low risk	Comment: same intervation for both groups
Blinding of outcome assessors (detection bias) -	Unclear risk	Comment: Insufficient detail reported
Incomplete outcome data (attrition bias)	Low risk	Comment: No loss reported

Selective reporting (reporting bias)	High risk	"HR and SBP were taken at the last minute of exercise"<br>Comment: Only systolic blood pressure outcome during exercise were reported in results sections.
Other bias	Low risk	Comment: Appears to be free of other bias

**Wingo et. al 2004**		
**Bias**	**Authors' judgement**	**Support for judgement**
Random sequence generation (selection bias)	Unclear risk	"Experimental trials (repeated-measures, random, crossover, double-blind design) were set under race conditions"<br>Comment: Insufficient detail reported
Allocation concealment(selection bias)	Unclear risk	"consumed pre-exercise and water via 2 water bottles consumed during the race"<br>Comment: Insufficient detail reported
Blinding (performance bias)	High risk	") no water during exercise (NE): water equal in volume to 2.8% body weight consumed pre-exercise and no water consumed during the race, (2) glycerol (G): mixture of water (equal in volume to 2.8% body weight) and glycerol (1 g/kg body weight ProHydrator, InterNutria Sports, Framingham, MA) consumed pre-exercise and water via 2 water bottles consumed during the race, and (3) water (W): water equal in volume to..."<br>Comment: Not possible to blind participants or researcher
Blinding of outcome assessors (detection bias) -	Unclear risk	Comment: Insufficient detail reported
Incomplete outcome data (attrition bias)	Low risk	Comment: No loss reported
Selective reporting (reporting bias)	Low risk	Comment: All pre-stated outcomes stated were reported

Other bias	Low risk	Comment: Appears to be free of other bias

**Zacharakis et al. 2013**		
**Bias**	**Authors' judgement**	**Support for judgement**
Random sequence generation (selection bias)	Unclear risk	"These 2 experiments were conducted 5–7 days apart with a random and counterbalanced order"<br>Comment: Insufficient detail reported
Allocation concealment(selection bias)	Unclear risk	"... the predicted fluid loss was replaced with water (21–22 °C). Water was provided with a syringe at least 5 min before the oral temperature"<br>Comment: Insufficient detail reported
Blinding (performance bias)	High risk	"for 60 min in a wheelchair with water intake (F) and without water intake (NF)."<br>Comment: Not possible to blind participants or researcher
Blinding of outcome assessors (detection bias) -	Unclear risk	Comment: Insufficient detail reported
Incomplete outcome data (attrition bias)	Low risk	Comment: No loss reported
Selective reporting (reporting bias)	Low risk	Comment: All pre-stated outcomes stated were reported
Other bias	Low risk	Comment: Appears to be free of other bias

SENSITIVITY ANALYZES

SENSITIVITY ANALYZES

1. **Meta-analysis performed without the cited studies in outcome "HR during exercise".**

Authors	TOTAL CI	Total Heterogeneity
Backhouse et. al 2007	-6.38 [-8.91, -3.85]	I^2 =75%
Macartney et. al 2019	-6.14 [-8.66, -3.63]	I^2 = 76%
Sanders et al. 1999	-6.10 [-8.65, -3.54]	I^2 = 76%
Wingo et. al 2004	-6.13 [-8.66, -3.61]	I^2 = 76%
Berkulo et al. 2015 a	-6.25 [-8.77, -3.73]	I^2 = 76%
Humm et al. 2008	-6.20 [-8.70, -3.69]	I^2 = 76%
Mendonca et. al 2013	-6.34 [-8.89, -3.79]	I^2 = 75%
Schoffstall et. al 2001	-6.16 [-8.73, -3.59]	I^2 = 76%
Armstrong et. al 1997 a	-6.28 [-8.82, -3.74]	I^2 = 76%
Armstrong et. al 1997 b	-5.77 [-8.24, -3.29]	I^2 = 74%
Berkulo et al. 2015 b	-6.26 [-8.77, -3.74]	I^2 = 76%
Hamilton et. al 1991	-6.05 [-8.68, -3.43]	I^2 = 76%
Hasegawa et al. 2006 a	-6.03 [-8.58, -3.47]	I^2 = 76%
Hasegawa et al. 2006 b	-6.63 [-9.03, -4.23]	I^2 = 70%
Heaps et. al 1994	-6.07 [-8.62, -3.52]	I^2 = 76%
Laurino et al. 2021	-6.32 [-8.85, -3.78]	I^2 = 76%
Lopez et al. 2011	-6.20 [-8.71, -3.68]	I^2 = 76%

Lynn et. al 2009 a	-5.99 [-8.64, -3.35]	$I^2 = 75\%$
Lynn et. al 2009 b	-5.56 [-7.42, -3.70]	$I^2 = 47\%$
McConell et al. 1999 a	-6.23 [-8.74, -3.71]	$I^2 = 76\%$
McConell et al. 1999 b	-6.22 [-8.74, -3.71]	$I^2 = 76\%$
Melo-Marins et. al 2018 a	-6.24 [-8.85, -3.62]	$I^2 = 76\%$
Melo-Marins et. al 2018 b	-6.44 [-8.97, -3.90]	$I^2 = 74\%$
Moreno et. al 2013 (II)	-6.20 [-8.75, -3.65]	$I^2 = 76\%$
Ribeiro et. al 2004 a	-6.38 [-8.89, -3.86]	$I^2 = 75\%$ (p=0.00)
Ribeiro et. al 2004 b	-6.43 [-8.93, -3.92]	$I^2 = 75\%$
Tripette et. al 2010 a	-6.40 [-8.93, -3.87]	$I^2 = 75\%$
Tripette et. al 2010 b	-6.22 [-8.76, -3.69]	$I^2 = 76\%$
Vanderlei et. al 2013 a	-6.26 [-8.81, -3.71]	$I^2 = 76\%$
Zacharakis et al. 2013 a	-6.28 [-8.92, -3.65]	$I^2 = 75\%$
Zacharakis et al. 2013 b	-6.04 [-8.61, -3.47]	$I^2 = 76\%$
Montain et al. 1992 a	-6.29 [-8.85, -3.74]	$I^2 = 76\%$
Montain et al. 1992 b	-6.10 [-8.64, -3.56]	$I^2 = 76\%$
Montain et al. 1992 c	-5.95 [-8.49, -3.42]	$I^2 = 75\%$
Moreno et. al 2012	-6.22 [-8.74, -3.69]	$I^2 = 76\%$
Vanderlei et. al 2013 b	-6.25 [-8.79, -3.71]	$I^2 = 76\%$
Lynn et al. 2009 a and Lynn et al. 2009 b	-5.19 [-7.00, -3.37]	$I^2 = 38\%$

2. Meta-analyzes performed without the cited studie in outcome "HRV on recovery"

Authors	TOTAL CI	Total Heterogeneity
Macartney et. al 2019	0.49 [0.30, 0.69]	$I^2 = 0\%$
Laurino et al. 2021	0.52 [0.32, 0.72]	$I^2 = 0\%$
Silva et. al 2022	0.46 [0.26, 0.66]	$I^2 = 0\%$
Castro-Sepúlveda et al. 2015	0.51 [0.32, 0.70]	$I^2 = 0\%$
Oliveira et al. 2011	0.49 [0.29, 0.68]	$I^2 = 0\%$
Peçanha et al. 2014	0.46 [0.27, 0.65]	$I^2 = 0\%$
Severeyn et al. 2016	0.47 [0.28, 0.66]	$I^2 = 0\%$
Teixeira et al. 2014	0.51 [0.32, 0.70]	$I^2 = 0\%$
Vanderlei et. al 2015	0.43 [0.23, 0.63]	$I^2 = 0\%$
Vianna et al. 2008	0.51 [0.31, 0.70]	$I^2 = 0\%$
Moreno et. al 2013 (II)	0.48 [0.28, 0.68]	$I^2 = 0\%$
Vanderlei et. al 2015 b	0.47 [0.27, 0.67]	$I^2 = 0\%$

3. Meta-analysis performed without the cited studies in outcome "SBP on recovery"

Authors	TOTAL CI	Total Heterogeneity
Humm et al. 2008	2.21 [0.03, 4.38]	$I^2 = 0\%$
Endo et. al 2012	2.27 [0.04, 4.49]	$I^2 = 0\%$
Vanderlei et. al 2013 a	2.34 [-0.18, 4.85]	$I^2 = 0\%$
Moreno et. al 2013 (II)	2.38 [0.17, 4.60]	$I^2 = 0\%$
Paula-Ribeiro et. al 2013	1.88 [-0.77, 4.53]	$I^2 = 0\%$
Teixeira et al. 2014	1.80 [-0.49, 4.10]	$I^2 = 0\%$
Moreno et. al 2012	2.96 [0.62, 5.31]	$I^2 = 0\%$
Vanderlei et. al 2013 b	2.12 [-0.13, 4.37]	$I^2 = 0\%$

4. Meta-analysis performed without the cited studies in outcome "DBP on recovery"

Authors	TOTAL CI	Total Heterogeneity

Humm et al. 2008	2.82 [-1.72, 7.35] (*p*=0.22)	I^2 = 62% (*p*=0.02)
Endo et. al 2012	2.94 [-1.66, 7.54] (*p*=0.21)	I^2 = 61% (*p*=0.02)
Vanderlei et. al 2013 a	2.80 [-1.91, 7.51] (*p*=0.24)	I^2 = 61% (*p*=0.02)
Moreno et. al 2013 (II)	3.24 [-1.33, 7.82] (*p*=0.16)	I^2 = 57% (*p*=0.03)
Paula-Ribeiro et. al 2013	0.63 [-3.08, 4.34] (*p*=0.74)	I^2 = 0% (*p*=0.92)
Teixeira et al. 2014	7.05 [5.21, 8.89] (*p*=0.00)	I^2 = 4% (*p*=0.39)
Moreno et. al 2012	3.24 [-1.33, 7.82] (*p*=0.16)	I^2 = 57% (*p*=0.03)
Vanderlei et. al 2013 b	3.06 [-1.54, 7.66] (*p*=0.19)	I^2 = 60% (*p*=0.02)
